# Impact of (k,t) sampling on DCE MRI tracer kinetic parameter estimation in digital reference objects

**DOI:** 10.1002/mrm.28024

**Published:** 2019-10-12

**Authors:** Yannick Bliesener, Sajan G. Lingala, Justin P. Haldar, Krishna S. Nayak

**Affiliations:** ^1^ Ming Hsieh Department of Electrical and Computer Engineering University of Southern California Los Angeles California

**Keywords:** brain tumor, data sampling, digital reference objects, dynamic contrast enhanced MRI

## Abstract

**Purpose:**

To evaluate the impact of (k,t) data sampling on the variance of tracer‐kinetic parameter (TK) estimation in high‐resolution whole‐brain dynamic contrast enhanced magnetic resonance imaging (DCE‐MRI) using digital reference objects. We study this in the context of TK model constraints, and in the absence of other constraints.

**Methods:**

Three anatomically and physiologically realistic brain‐tumor digital reference objects were generated. Data sampling strategies included uniform and variable density; zone‐based, lattice, pseudo‐random, and pseudo‐radial; with 50‐time frames and 4‐fold to 25‐fold undersampling. In all cases, we assume a fully sampled first time frame, and prior knowledge of the arterial input function. TK parameters were estimated by indirect estimation (i.e., image‐time‐series reconstruction followed by model fitting), and direct estimation from the under‐sampled data. We evaluated methods based on the Cramér‐Rao bound and Monte‐Carlo simulations, over the range of signal‐to‐noise ratio (SNR) seen in clinical brain DCE‐MRI.

**Results:**

Lattice‐based sampling provided the lowest SDs, followed by pseudo‐random, pseudo‐radial, and zone‐based. This ranking was consistent for the Patlak and extended Tofts model. Pseudo‐random sampling resulted in 19% higher averaged SD compared to lattice‐based sampling. Zone‐based sampling resulted in substantially higher SD at undersampling factors above 10. CRB analysis showed only a small difference between uniform and variable density for both lattice‐based and pseudo‐random sampling up to undersampling factors of 25.

**Conclusion:**

Lattice sampling provided the lowest SDs, although the differences between sampling schemes were not substantial at low undersampling factors. The differences between lattice‐based and pseudo‐random sampling strategies with both uniform and variable density were within the range of error induced by other sources, at up to 25‐fold undersampling.

## 1 INTRODUCTION

Dynamic contrast enhanced MRI (DCE‐MRI) of the brain provides a powerful tool to non‐invasively assess neurovascular parameters, such as permeability of the brain–blood barrier (*K* ^trans^), plasma (*v*_p_), and interstitial volume fraction (*v*_e_). Brain–blood barrier dysfunction with abnormal permeability has been shown to occur in multiple pathologies, including Alzheimer's disease, multiple sclerosis, acute ischemic stroke, and brain tumors.^1^, ^2^ For brain metastases, quantification of permeability as well as perfusion shows potential to not only classify tumors, but moreover to predict, guide, and validate treatment response and success rate. Although much of the required information is available through images of tumor morphology on conventional post‐contrast T_1_‐weighted MRI, functional changes that can be monitored with DCE MRI happen on a shorter time scale. This makes DCE MRI a candidate to monitor early response to therapy and to potentially alter treatment to mend side‐effects or to opt for a more aggressive treatment and potentially enhance chances of survival.^3^

For routine clinical use, DCE‐MRI measurements of tracer‐kinetic (TK) parameters have to be accurate, precise, and reproducible.^3^, ^4^, ^5^ The precision of DCE MRI imaging has been the subject of a long history of studies.^6^, ^7^, ^8^, ^9^ Among other dependencies, precision of TK parameter maps depends highly on the underlying (k,t) data sampling pattern and the ability to find a suitable estimator to reconstruct TK parameters from a given set of data samples.^10^ Undersampling within each time frame is inevitable to achieve desired temporal resolution to reliably capture bolus arrival, accumulation, and wash‐out, while still maintaining a clinically relevant FOV at high spatial resolution.^1^, ^2^

Numerous (k,t) sampling strategies have been proposed for dynamic MRI in general (i.e., not specific to DCE‐MRI). For Cartesian sampling, these strategies could broadly be classified into zone‐based,^11^, ^12^, ^13^ lattice‐based,^14^, ^15^, ^16^ and random sampling with uniform density^17^ or variable density.^18^, ^19^, ^20^ Several of these approaches are based on specific imaging or modeling assumptions (e.g., x‐f support),^21^, ^22^, ^23^ and are tailored to the needs of a specific constrained reconstruction procedure. A variety of methods have also been proposed that formulate k‐ and (k,t)‐space sampling design as an optimization problem.^21^, ^23^, ^24^, ^25^, ^26^, ^27^, ^28^, ^29^, ^30^, ^31^, ^32^, ^33^, ^34^, ^35^, ^36^

Inherent to DCE‐MRI is a very high data redundancy in the mapping from multi‐coil, multi‐frame k‐space data to a few static TK parameter maps. This data redundancy can be exploited with a maximum‐likelihood‐like estimator^37^ that relaxes the requirements on the sampling strategy. Hence, it remains crucial to gain insight how the achievable precision of TK parameters scales for different sampling strategies as the undersampling rates increase to allow for a clinical high‐resolution whole‐brain DCE‐MRI.

In this work, we compare (k,t) sampling patterns in a framework that assumes TK model consistency without making additional assumptions about the object (e.g., that it is smooth, has limited support, etc.). We use pathologically and anatomically realistic digital reference objects and use both Cramér‐Rao bound (CRB) analysis and Monte‐Carlo simulation to evaluate TK parameter variances. We compare 8 commonly used (k,t) data sampling patterns over a range of undersampling factors and SNR levels relevant to high‐resolution whole‐brain DCE‐MRI.

## 2 METHODS

### 2.1 Digital reference object

**Figure 1:**
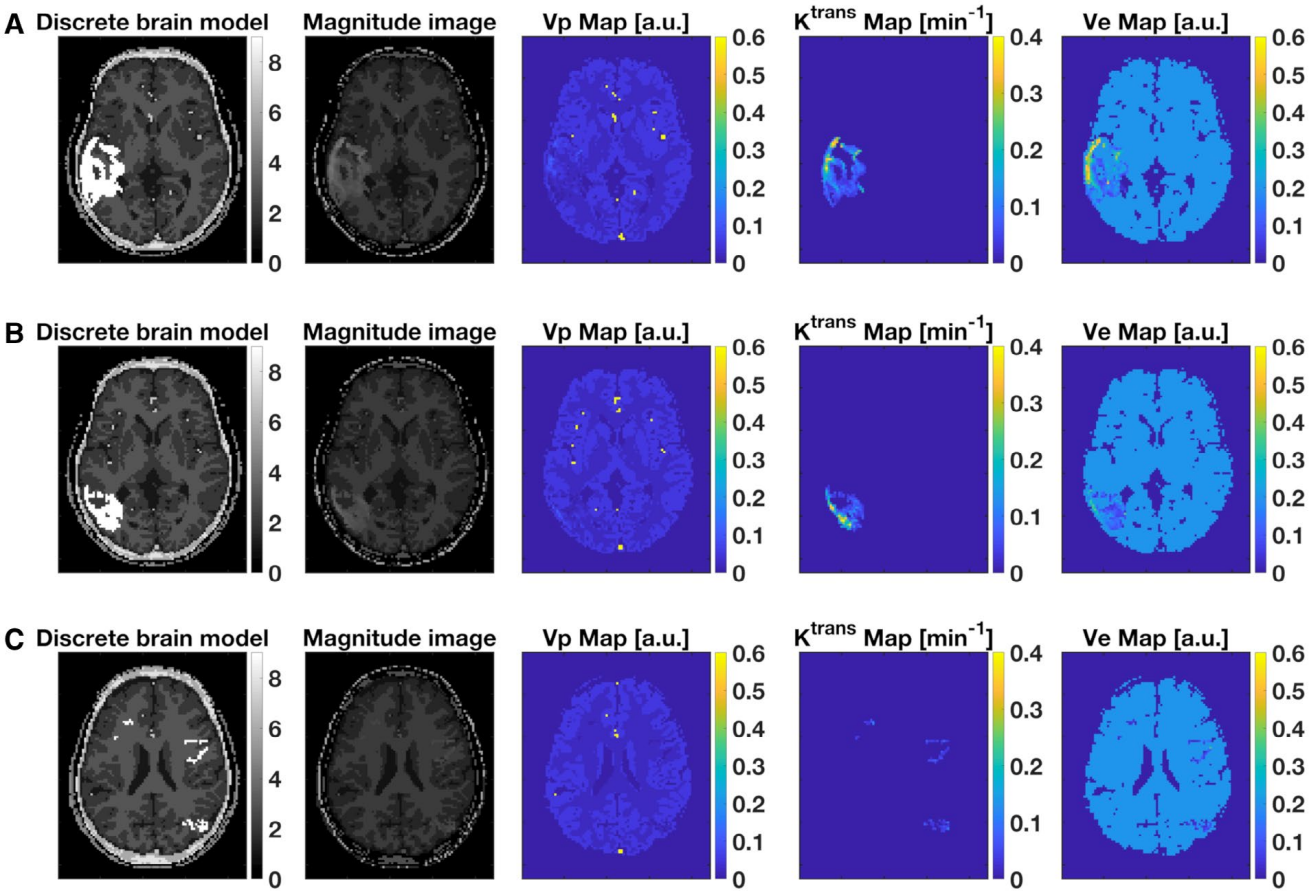
Three DROs were used to test for robustness of the results: Brain slice with large tumor with enhancing rim (A), brain slice with smaller tumor (B), and brain slice with metastases (C). These were formed by inserting previously developed brain tumor DCE‐MRI reference objects^38^ into brain data from subject 4 of the BrainWeb Database.^68^ Through this process, additional DROs can be easily created

The optimal sampling strategy is expected to depend on the image characteristics and the specific details of the forward model. As a result, we conducted our analysis using realistic digital references objects (DROs) that are typical of the protocol used at our imaging center. Pathologically and anatomically realistic DROs^38^ were taken to be the ground‐truth. Axial 2D DRO slices were used to evaluate the impact of (k,t) sampling on 3D DCE‐MRI, where the fully sampled readout direction is superior‐inferior. Cartesian 2D phase encoding is performed in the axial *k_y_*‐*k_z_* plane, and the impact of sampling can be adequately studied with 2D axial DROs in the y‐z plane. All brain slices were cropped to contain as little space around the head as possible and had 106 × 88 pixels which corresponds to an approximate voxel size of 4 mm × 4 mm × 5 mm. To cover a broader range of tumors that are likely to be found in clinical exams, 3 brain DROs with one large tumor, one small tumor, and one metastasized tumor were used. Tumor data originated from high grade glioma patients. The DROs are shown in Figure 1.

Each tissue was then simulated at 3T using the proton density ρ, precontrast spin‐lattice relaxation time *T*_10_, plasma volume *v*_p_, interstitial volume fraction *v*_e_, and volume‐transfer constant *K* ^trans^ listed in Supporting Information Table S1. All DROs were based on the following experimental parameters: pre‐contrast white‐matter SNR = 10–100, 25° flip angle, 50 time frames, 5 s temporal resolution, and a 5 ms repetition time (i.e., 1000 phase encodes per time frame). These are typical parameters for brain DCE‐MRI and are within the recommend range of the current RSNA‐QIBA DCE‐MRI Profile.^39^

### 2.2 Data model

DCE MRI monitors bolus arrival, accumulation, and wash‐out over time in a given field‐of‐view (FOV). Specifically, the DCE MRI experiment can be modelled mathematically as

(1) $$k_{t}=\text{UFS}\Phi_{t}\left(\theta \right)+n_{t},$$

where **k** *_t_* denotes samples in (k,t) space, U the undersampling operator, F the Fourier operator, S coil sensitivity encoding, and Φ*_t_* incorporates Bloch simulation and TK modeling to generate the signal intensity at time *t* for a given set of tracer‐kinetic parameters *θ*. **n** *_t_* is additive complex, circularly symmetric Gaussian noise.^40^ The signal intensity was simulated for a spoiled gradient echo (SPGR) pulse sequence assuming the fast exchange limit for conversion from concentration time curves to longitudinal relaxation. To investigate the generalizability of the results across different models, we generated concentration time curves following the linear Patlak model as well as the nonlinear extended Tofts model.^41^ For both TK models, this results in a nonlinear mapping from the TK parameters to the measured complex k‐space data. A population‐based arterial input function was chosen for this simulation.^42^ Coil sensitivities for an 8‐channel head array and the noise covariance matrix were taken from measurements of a typical brain tumor patient undergoing a clinical DCE MRI exam at our institution (3T, HD23, GE Healthcare, Waukesha, WI).

### 2.3 Data sampling patterns

**Figure 2:**
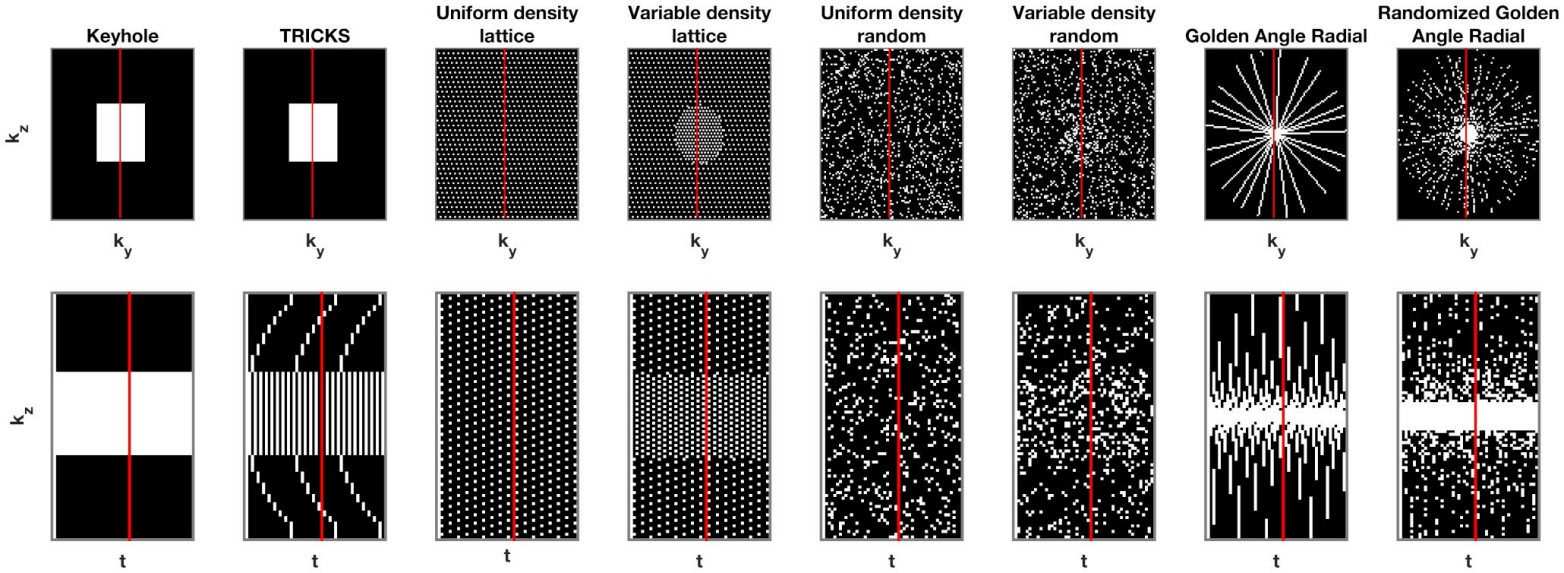
Illustration of data sampling strategies for 9‐fold undersampling. Shown are the *k_y_*, *k_z_* phase‐encoding plane for the 29th time frame (top row), and the *k_z_*‐t plane for the central *k_y_* (bottom row). White dots indicate phase‐encodes that are acquired (i.e., a whole line k‐space along *k_x_*). Black indicate phase‐encodes that are not acquired. Left‐to‐right: Keyhole, TRICKS, lattice with uniform density, lattice with variable density, random with uniform density, and random with variable density, pseudo‐radial with Golden Angle increments (GAR) and randomized pseudo‐radial with Golden Angle increments (RGAR). Each sampling pattern had a fully sampled first time frame and varied for all subsequent time frames, as shown in Supporting Information Video S1

Eight different sampling patterns were compared and are shown in Figure 2 and Supporting Information Video S1. For all sampling patterns, the first time frame was fully sampled. Although many different sampling strategies exist for dynamic MRI exams, many of these combine ideas of zone‐based, lattice, random sampling, both in uniform and variable density variants, or (pseudo) radial sampling. We compared each of these sampling pattern classes with each other. In this study, zone‐based (k,t) space sampling is represented by Keyhole sampling^43^ and TRICKS.^12^ In contrast to Keyhole sampling,^43^ TRICKS updates the outer k‐space on a regular basis. Therefore, the task of filling in missing (k,t) space samples becomes an interpolation rather than an extrapolation problem which is intrinsically better posed.^30^, ^44^, ^45^ For lattice‐based sampling, a (k,t) lattice was used with uniform (UD) and variable lattice density (VD). Breuer et al^46^ introduced CAIPIRINHA‐type lattice sampling whose structure is generated by replication of elementary cells of size *R* × *R* in the 2D phase‐encoding plane for a given sampling reduction factor *R*. The CAIPIRINHA‐type reduction factor is specified by

(2) $$R_{\text{CAIPI}}=R_{y}\;\times \;R_{z}^{\left(\Delta \right)},$$

with *R*_y_ and *R*_z_ as reduction factors in the two phase‐encoding directions, and Δ the circular shift between two consecutive rows of the lattice elementary cell. Breuer et al^46^ found a lattice generated according to $R_{\text{CAIPI}}=1\;\times R^{\sqrt{R}}$ to yield among the lowest g‐factor for 3D (static) brain imaging. Following this notation, the elementary cells of the 3D phase‐encoding lattice (*k_y_* × *k_z_* × *t*) in this study are chosen according to

(3) $$R_{\text{CELL}}=1\times R^{\sqrt{R}}\times 1^{1}.$$

The lattice structure is built from the elementary cells by replication of the elementary cells in linear order along the two phase‐encodes *k_y_*, *k_z_*, and time *t* before cropping the lattice to the desired k‐space dimensions. Analogously, random sampling was implemented with uniform and variable density in (k,t) space. For each of these Cartesian sampling patterns, the number of samples per frame and region of sampling density are approximately identical for every frame. We further included pseudo radial sampling with golden angle increments (GAR) and its randomized derivative in our analysis (RGAR).^20^ For both variants, the spokes are generated center‐out with counter‐clockwise Golden Angle increments. For RGAR, the samples along a spoke are accepted by chance with probability 0.3. These pseudo radial sampling patterns replace non‐Cartesian sampling locations by the nearest neighbor on a Cartesian grid. This allows pseudo radial sampling patterns to be analyzed in the same pipeline with the Cartesian sampling patterns. Every sampling trajectory in this study assumes instantaneous sampling within each time frame.

### 2.4 Monte‐Carlo simulation

We used Monte‐Carlo simulations to quantify the precision of TK parameter fitting. We evaluated efficiency of direct reconstruction^37^ and SENSE reconstruction with subsequent TK parameter fitting of concentration time curves. Direct reconstruction poses the estimation of TK parameters as optimization problem of the form

(4) $${\text{argmin}}_{\theta }\|C^{-1}\left(\text{UFS}\Phi_{t}\left(\theta \right)-k_{t}\right){\|}_{F}^{2}.$$

In the weighted least squares (WLS) variant, *C* is chosen to be the Cholesky decomposition of the noise covariance matrix. In the ordinary least squares (OLS) approach *C* is set to identity. In both cases the optimization problem is solved using nonlinear conjugate gradient descent.

We did not apply any spatial or temporal constraint during SENSE reconstruction. Monte‐Carlo simulations were performed over a pre‐contrast white matter SNR range from 10–100. The goal was to determine the operational minimum SNR for each method. This range covers best case and clinically realistic scenarios down to noise levels that make TK parameter estimation practically infeasible. For each SNR level, we used 100 noise realizations that were found to be sufficient for the variance estimates to stabilize.

### 2.5 Data analysis

We compared the Cramér‐Rao bound (CRB) for the common TK parameters of the extended Tofts and the Patlak model (i.e., *v*_p_ and *K* ^trans^) for a broad range of undersampling factors. The CRB gives a lower bound on the covariance of any unbiased estimator for the desired parameter vector,^47^ and has been widely used to optimize MRI experiment design^7^, ^28^, ^36^, ^48^, ^49^

(5) $$\text{Cov}\left(\theta \right)\geq J^{-1}\left(\theta \right).$$

Here, J(θ) is the Fisher information matrix (FIM). The corresponding unbiased estimator that meets this bound tightly is called efficient. If the FIM is singular, this indicates that the CRB is near‐infinite for certain parameters (i.e., those parameters are not able to be estimated accurately), which is undesirable. Hence, from a measurement point of view these settings should be avoided if possible.^50^ An example of such experiment setup is given by Keyhole sampling strategies.^43^ In Keyhole sampling, the first pre‐contrast time frame is fully sampled, whereas all subsequent frames trade low‐spatial resolution for higher temporal resolution. Because the pre‐contrast frame does not contain any information about TK parameters, limited high spatial frequency information is available for TK the parameter maps.

The CRB values were computed for the entire image but were analyzed within regions‐of‐interest (ROIs) tightly drawn around all tumors. We also compared spatial maps of the CRB at an undersampling factor of 16. To be able to compare all sampling patterns at the same undersampling factor, the figures of merit for lattice sampling with variable density were linearly interpolated to match the nominal undersampling factors.

## 3 RESULTS

**Figure 3:**
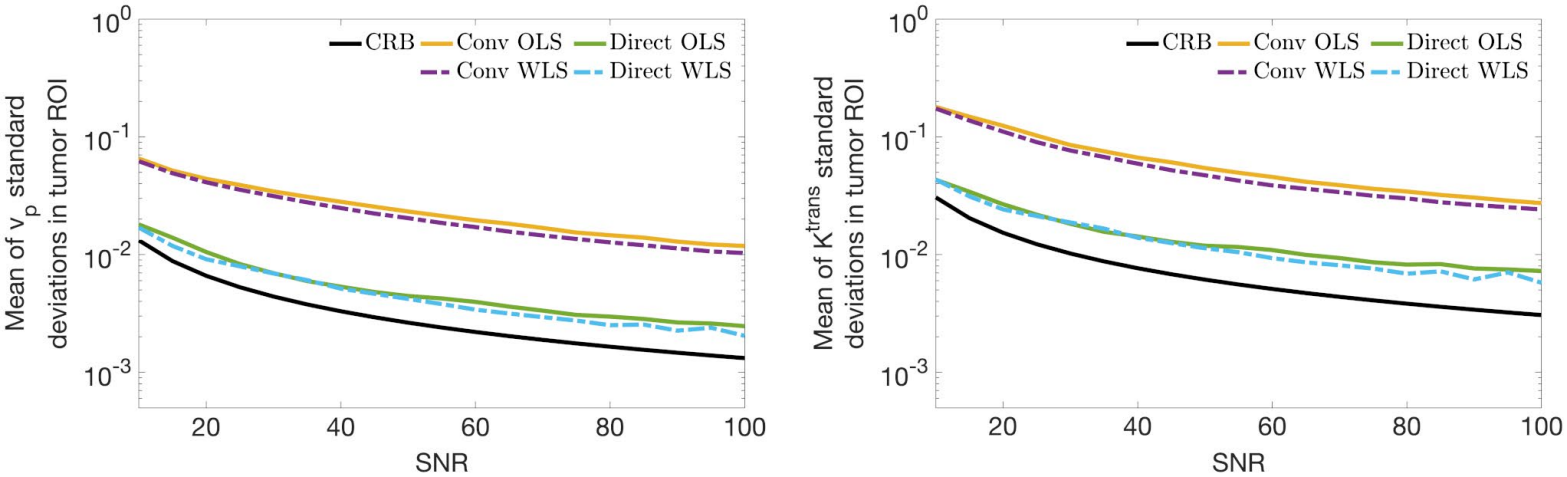
Estimator performance as a function of SNR for both *v*_p_ (left column) and *K* ^trans^ (right column) of the extended Tofts model: Comparison of SDs predicted by CRB with SDs computed by Monte‐Carlo simulation with conventional (conv) and direct reconstruction with (WLS) and without (OLS) accounting for correlated, non‐isotropic noise for uniform random sampling with R = 4 and uniform density undersampling

Figure 3 summarizes estimator performance for the extended Tofts model as a function of SNR and undersampling factor. The CRB is compared with empirical SDs from Monte‐Carlo simulations with conventional and direct reconstruction both with (WLS) and without (OLS) accounting for correlated, non‐isotropic noise. For both TK parameters, SDs of direct parameter estimation in both formulations were always within 2.5 times the CRB. Conventional indirect reconstruction results in SDs of about one order of magnitude above the CRB. For both reconstruction approaches, the weighted least squares formulation leads to small improvements in achievable SDs.

**Figure 4:**
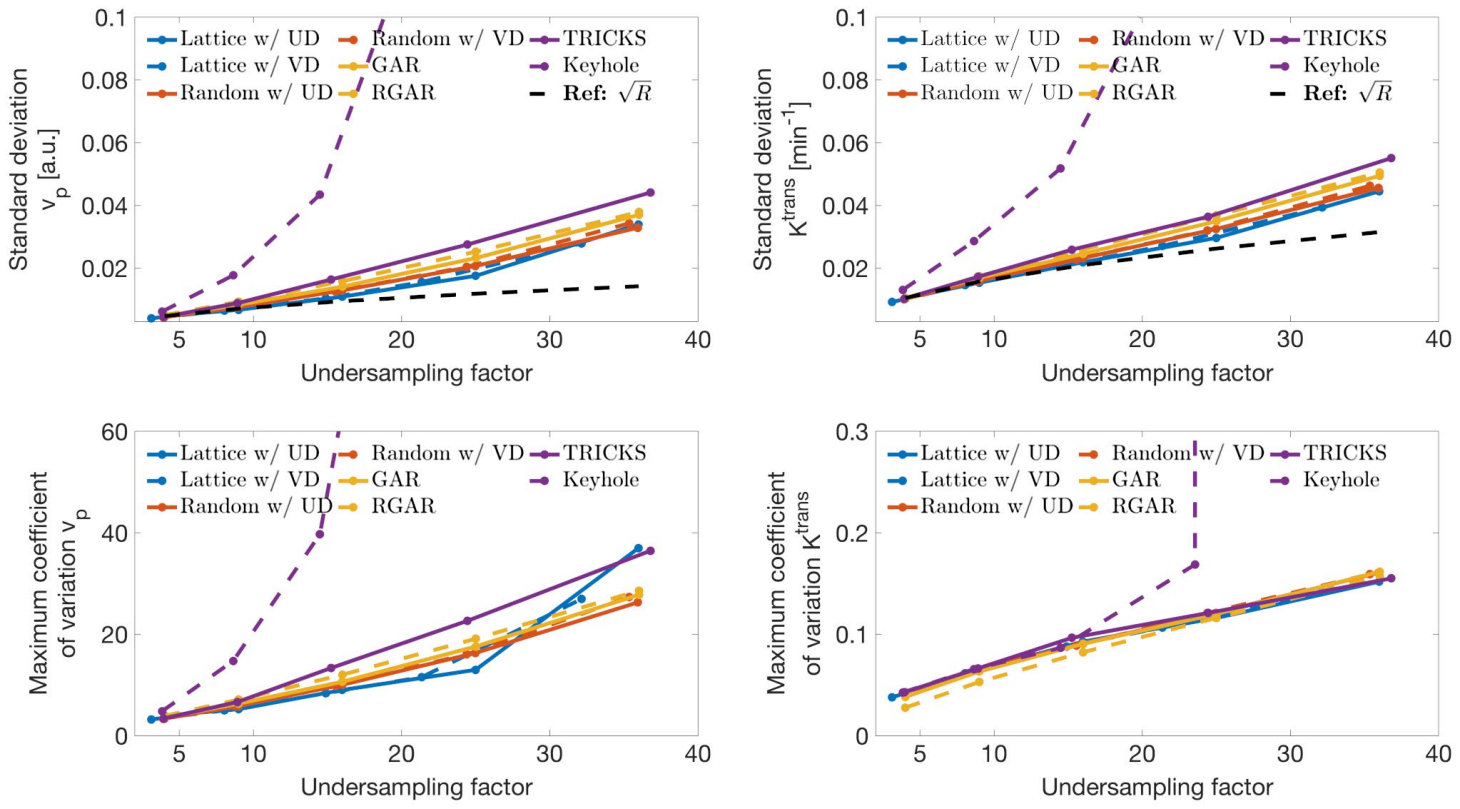
Comparison of sampling patterns as a function of undersampling factor for the estimation of *v*_p_ (left column) and *K* ^trans^ (right column) of the extended Tofts model. CRB‐predicted SDs averaged over brain tumor regions are shown in the top row and maximum coefficient of variation (i.e., SD divided by true parameter) in the bottom row. For all sampling patterns except Keyhole sampling, the averaged SDs for both TK parameters scale approximately with the square root of the undersampling factor at low undersampling factors. As the undersampling factor is increased, the scaling behavior deviates from this rule indicating an increased interaction between the undersampling and the coil geometry

Figure 4 shows CRB‐based predictions of the average SD and maximum coefficient of variation of TK parameters *v*_p_ and *K* ^trans^ for the extended Tofts model across the three tumor ROIs. For all sampling patterns except Keyhole sampling, the averaged SDs for both TK parameters scale approximately with the square root of the undersampling factor, which indicates the loss in measurement precision to be dominated by fewer measurements and shorter acquisition time.^51^ As the undersampling factor is increased, the scaling behavior deviates from this rule indicating an increased interaction between the undersampling and the coil geometry.^52^ At low undersampling rates (i.e., R = 4), lattice sampling with variable density and both variants of random sampling achieve lower (average) SDs than lattice sampling with uniform density for *v*_p_ whereas they are all approximately equal for *K* ^trans^. At all higher undersampling rates (i.e., R ≥ 9), lattice sampling with uniform density outperforms all other sampling strategies in this comparison. Although Keyhole sampling achieves SDs comparable to those of the other seven sampling strategies in this comparison, the SDs of Keyhole sampling lie well above the all other sampling patterns at R ≥ 9.

**Figure 5:**
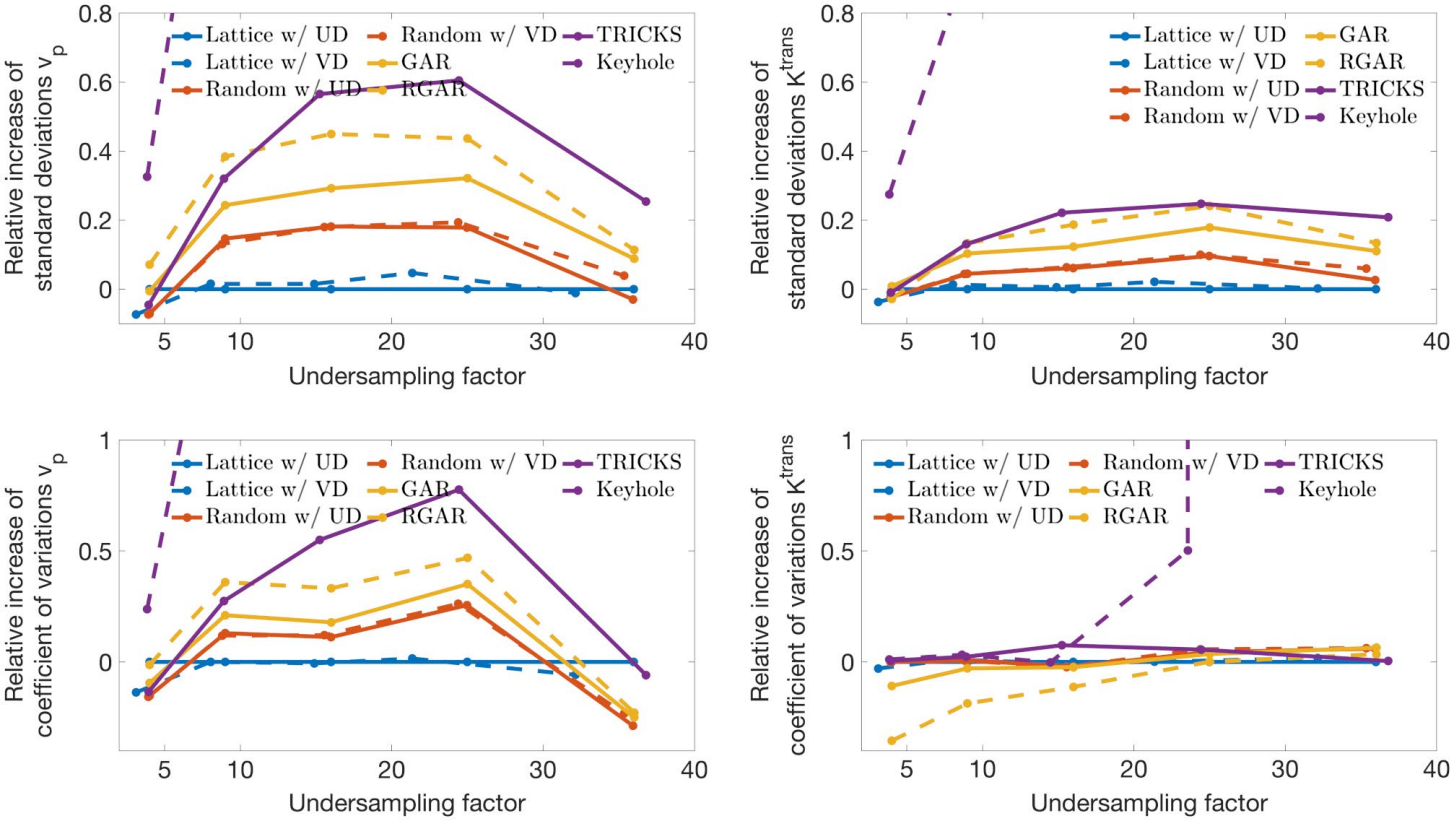
Relative comparison of sampling patterns as a function of undersampling factor for the estimation of *v*_p_ (left column) and *K* ^trans^ (right column) for the extended Tofts model. Relative increase in SD bounds averaged across the tumor ROI (top row) and maximum coefficients of variation across the tumor ROI (bottom row) are shown with respect to figures of lattice with UD. Although random sampling with uniform and variable density perform best at R = 4, lattice sampling leads to lowest average SD for both *v*_p_ and *K* ^trans^ compared to uniform sampling at higher undersampling factors. For both lattice and random sampling, the uniform density variants perform comparable to their variable density counterparts

Figure 5 shows the increase in the CRB‐based predictions of the SD and coefficient of variation (relative to lattice sampling with uniform density) as a function of undersampling factor for the extended Tofts model. Overall, the relative differences in achievable SDs between sampling patterns are higher for *v*_p_ than for *K* ^trans^. There is an increase in average SDs of up to 19% for *v*_p_ and 10% for *K* ^trans^ when random instead of lattice sampling is applied. For both lattice and random sampling, there is little difference among their respective uniform and variable density layouts. For pseudo radial sampling with GAR, randomization leads to an increase of SD of up to 14% for *v*_p_ and up to 7% for *K* ^trans^. With regard to the maximum coefficient of variation, the relative ranking between different sampling patterns remains approximately identical for *v*_p_ up to R = 25 compared to the ranking seen for the averaged SDs. For *K* ^trans^, the maximum coefficients of variation obtained by TRICKS, random sampling with uniform and variable density, lattice sampling with uniform and variable density, and Golden Angle radial are roughly identical across all undersampling rates. At lower undersampling rates, i.e., R < 25, randomized Golden Angle pseudo‐radial sampling achieves lower maximum coefficient of variation compared to all other sampling patterns.

**Figure 6:**
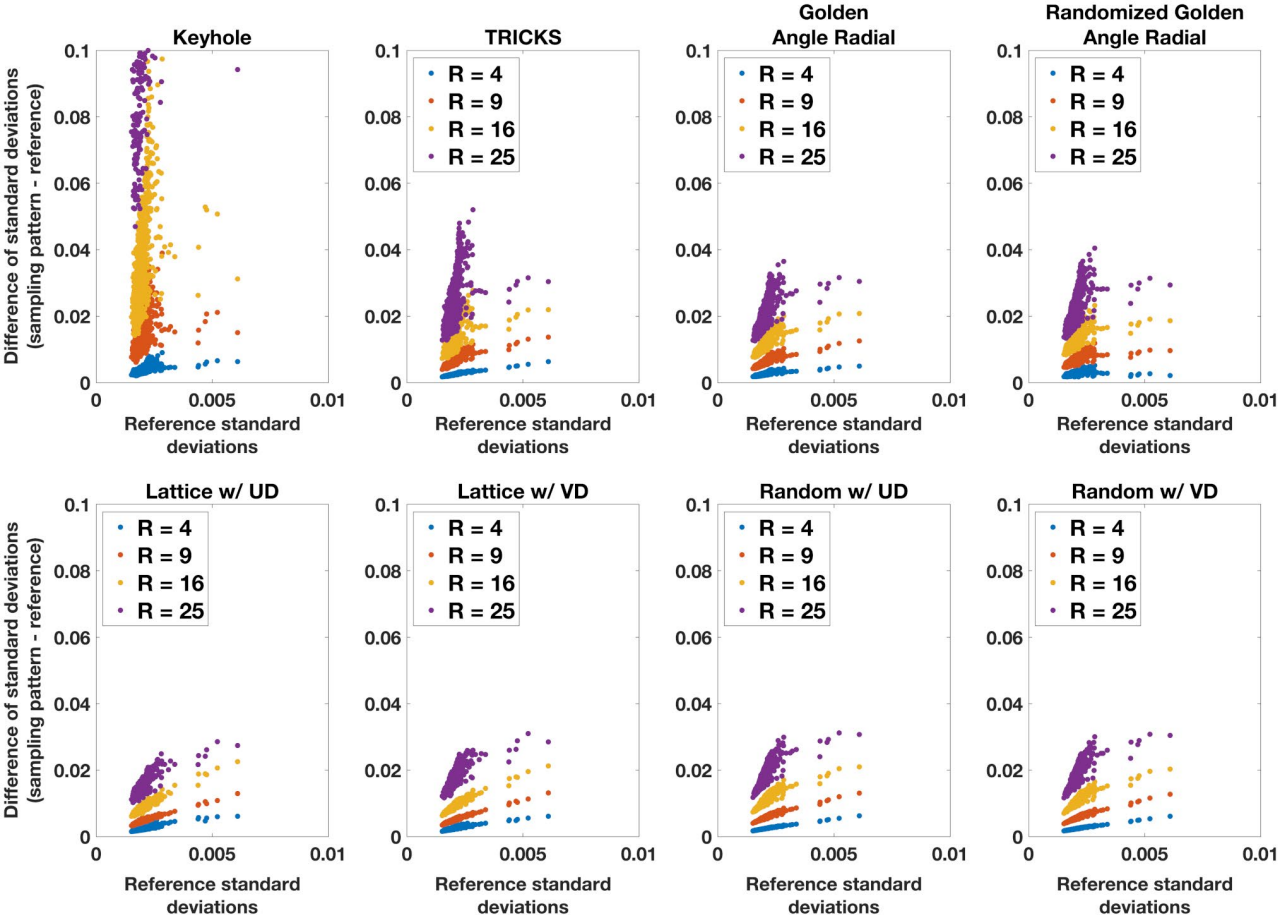
CRB‐predicted SD bounds for the extended Tofts tracer‐kinetic parameter *v*_p_ within brain tumor regions of interest, as a function of sampling strategy and undersampling factor. Fully sampled data is chosen as reference. Top row left to right: Keyhole sampling, TRICKS, Golden Angle radial, and randomized Golden Angle radial. Bottom row left to right: lattice with uniform (UD) and variable density (VD), random sampling with uniform (UD). and variable density (VD). Different undersampling factors are indicated by color. Bounds are shown for SNR = 30

**Figure 7:**
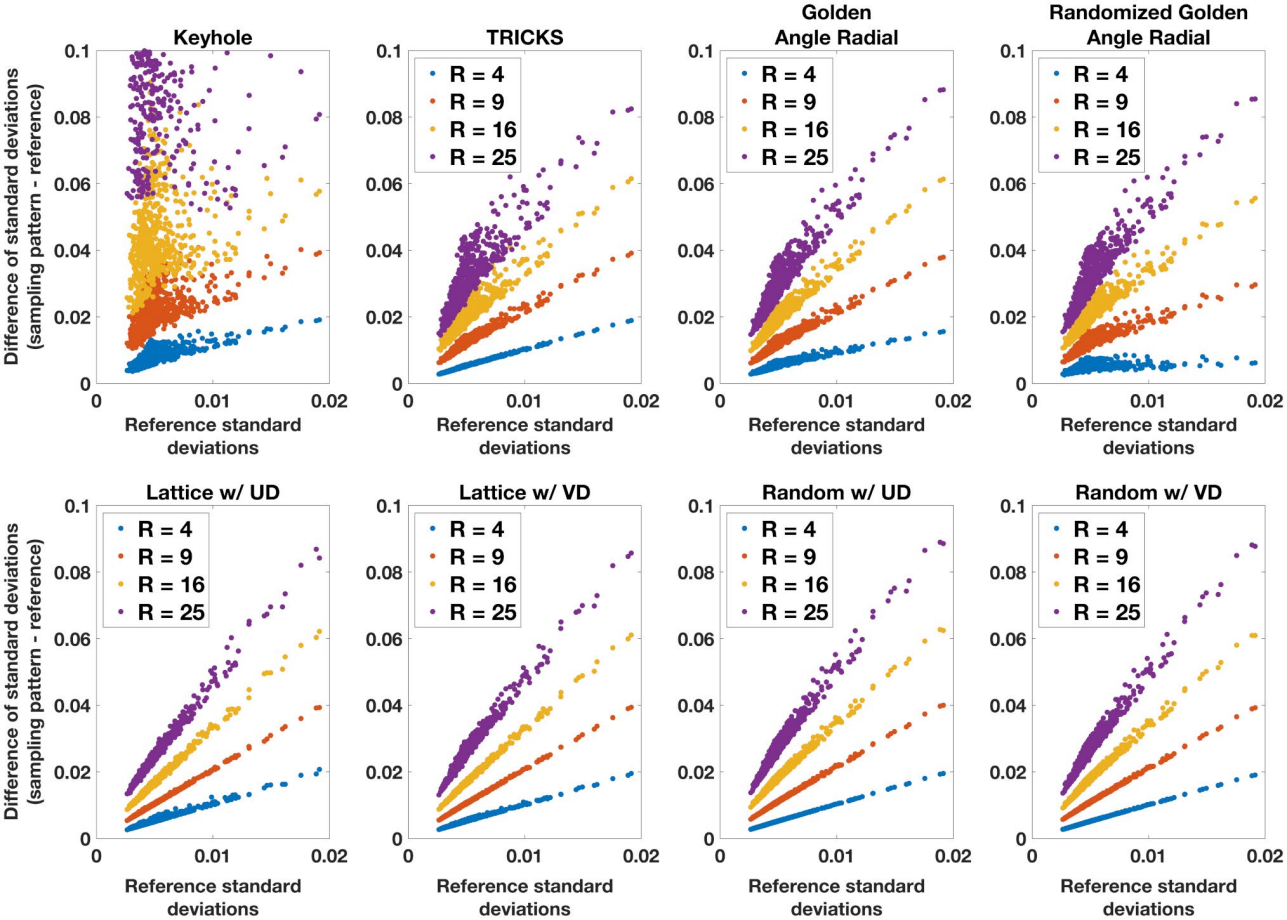
CRB‐predicted SD bounds for the extended Tofts tracer‐kinetic parameter *K* ^trans^ within brain tumor regions of interest, as a function of sampling strategy and undersampling factor. Fully sampled data is chosen as reference. Top row left to right: Keyhole sampling, TRICKS, Golden Angle radial, and randomized Golden Angle radial. Bottom row left to right: lattice with uniform (UD) and variable density (VD), random sampling with uniform (UD), and variable density (VD). Different undersampling factors are indicated by color. Bounds are shown for SNR = 30

Figures 6 and 7 compare CRB‐based predictions of the SDs for TK parameters *v*_p_ and *K* ^trans^ of the extended Tofts model across all tumor ROIs for the DROs in Figure 1 as a function of sampling strategy and undersampling factor. All comparisons are performed with respect to fully sampled data. Confirming findings of Figures 4 and 5, both lattice and random sampling strategies perform similarly well at R = 4. With higher undersampling factors, lattice sampling with uniform density gives lowest SDs. For the estimation of *K* ^trans^, the plots in Figure 7 show that lattice and random sampling strategies lead to an approximately linear increase in the observed SD (i.e., a higher SD in the fully sampled reference also entails a higher increase of the SD because of undersampling). The slope of this effect increases with undersampling factor. TRICKS and GAR share a similar behavior at low R, while RGAR is overall best performing at R = 4. At higher undersampling factors, Keyhole, TRICKS, GAR, and RGAR show an increased number of tumor voxels with low SD in the fully sampled case but high (super‐linear) increase of SDs because of undersampling.

**Figure 8:**
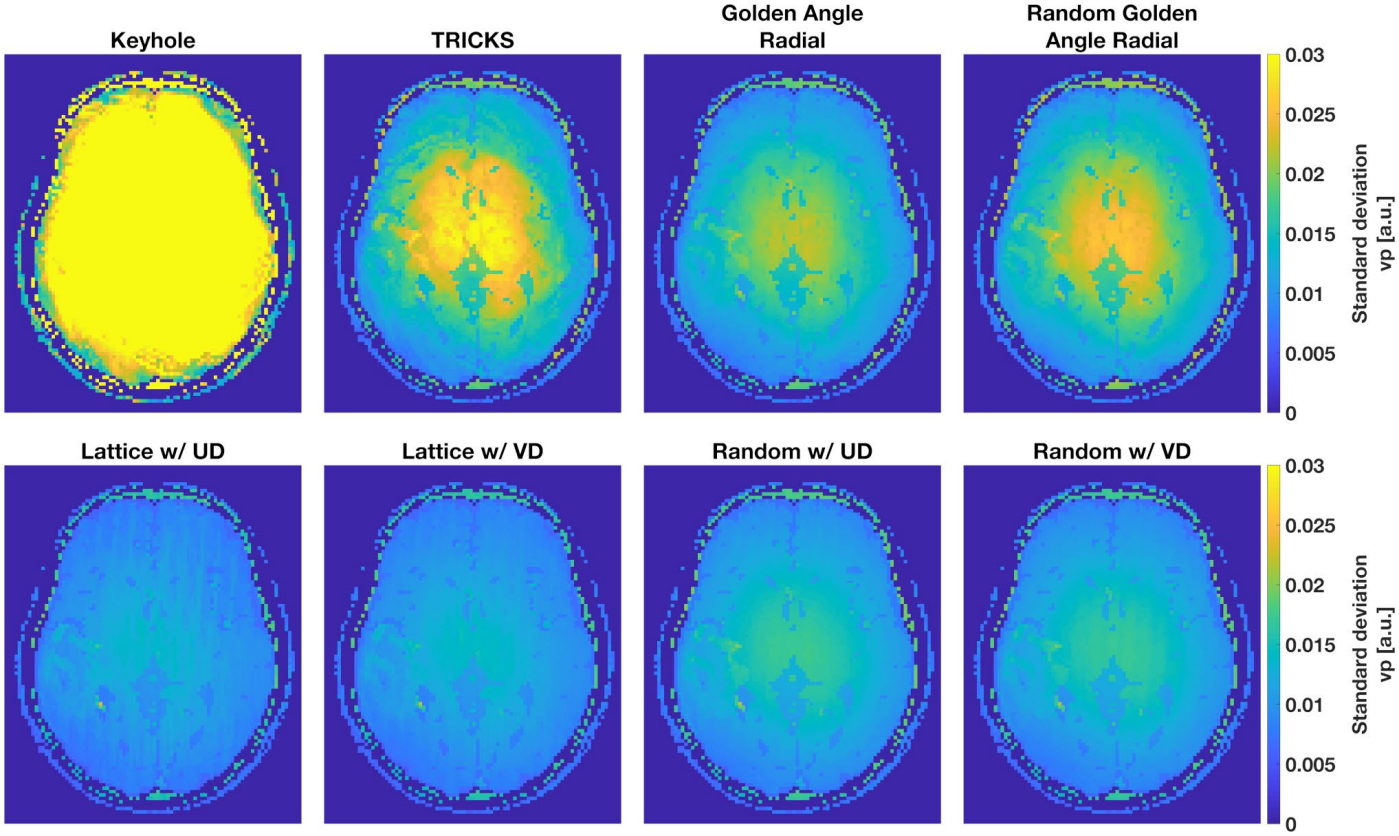
Visualization of extended Tofts *v*_p_ parameter SDs predicted by CRB for different sampling patterns at undersampling factor 16 for the first DRO in Figure 1. Top row left to right: Keyhole sampling, TRICKS, Golden Angle radial, and randomized Golden Angle radial. Bottom row left to right: lattice with uniform (UD) and variable density (VD), random sampling with uniform (UD), and variable density (VD). All sampling patterns show central enhancement of SD bounds for both TK parameters

**Figure 9:**
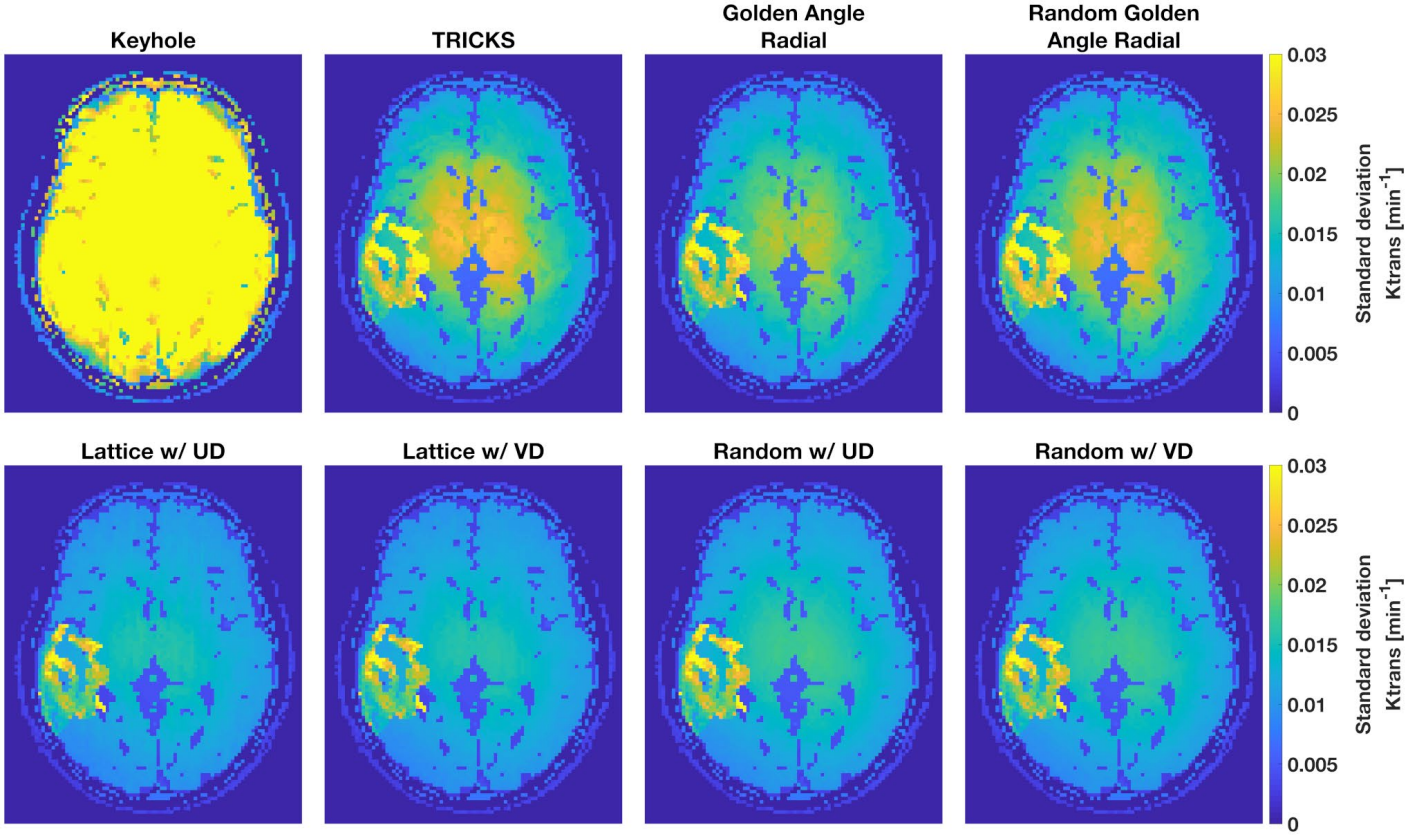
Visualization of extended Tofts *K* ^trans^ parameter SDs predicted by CRB for different sampling patterns at undersampling factor 16 for the first DRO in Figure 1. Top row left to right: Keyhole sampling, TRICKS, Golden Angle radial and randomized Golden Angle radial. Bottom row left to right: lattice with uniform (UD) and variable density (VD), random sampling with uniform (UD), and variable density (VD). All sampling patterns show central enhancement of SD bounds for both TK parameters

Figures 8 and 9 illustrate spatial distributions of CRBs for the extended Tofts model computed for all sampling patterns for an undersampling factor of 16. All sampling patterns show enhancement of the bounds in the center of the slice that coincides with the tissue region of least sensitivity of the pick‐up coil array. Again, Keyhole, TRICKS, and pseudo‐radial sampling show the highest bounds. TRICKS sampling exhibits a spiral‐like pattern in the predicted SD maps. An SNR map for the fully sampled multi‐channel coil configuration showing a similar swirl‐like pattern can be found in Supporting Information Figure S9. For both lattice sampling patterns, the CRBs in Figures 8 and 9 show stripes of enhanced SD.

**Figure 10:**
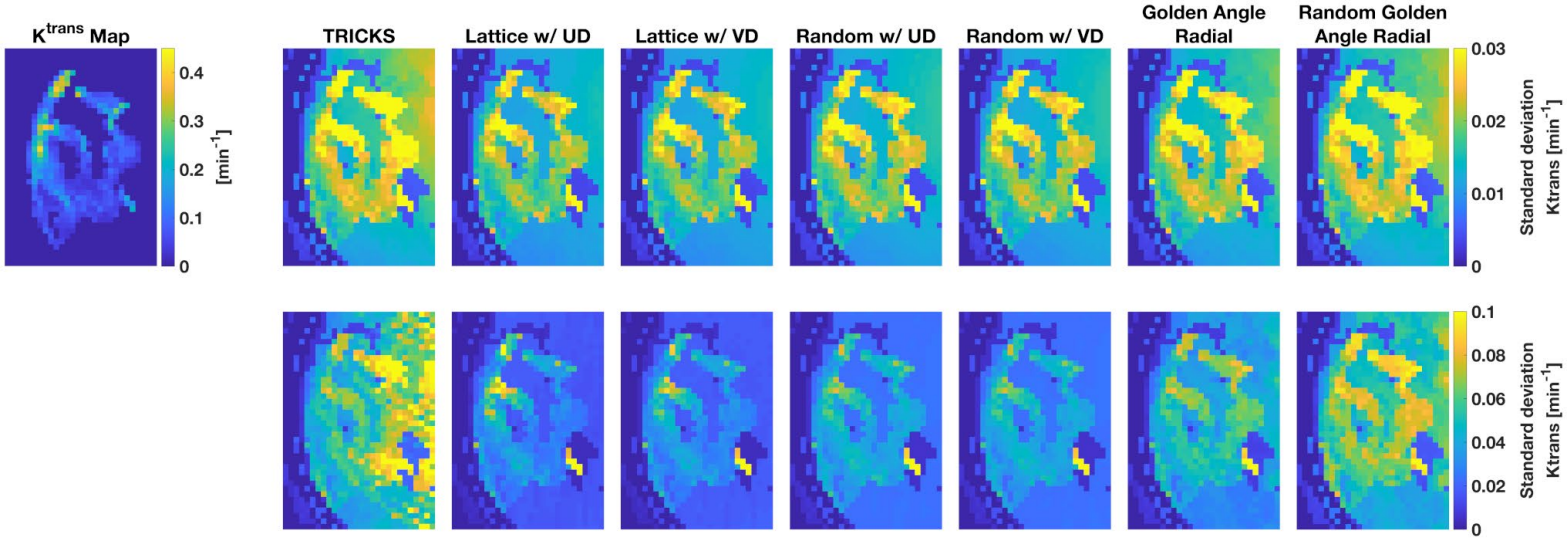
Visualization of extended Tofts *K* ^trans^ SDs predicted by CRB for different sampling patterns at undersampling factor 16 for the tumor region of the first DRO in Figure 1. Top row: CRB accounts for measured coil sensitivities from an 8‐channel head array. Bottom row: CRB is computed for a single channel with uniform sensitivity

Figure 10 shows the spatial distribution of CRBs for *K* ^trans^ of the extended Tofts model zoomed into the tumor region of the first DRO in Figure 1. The bounds are shown for a sample undersampling factor of 16 with and without accounting for coil sensitivities in the CRB computation. When the impact of coil sensitivities is disregarded, SDs throughout the tumor are dominated by proximity to the brain center. Decoupling this effect by computing the CRB for a single channel with uniform coil sensitivity reveals lattice and random sampling to have comparable ability to detect tumor heterogeneity. For TRICKS, GAR, and RGAR, the spatial distribution of SDs is substantially different from lattice and random sampling with higher SD toward the center of the field‐of‐view.

Figures 3, 4, 5, 6, 7, 8, 9, 10 are replicated for the Patlak model and are shown in the supporting information as Supporting Information Figures S1–S8. As opposed to the TK parameter estimation for the extended Tofts model, direct reconstruction in WLS formulation provides an efficient estimator for the (linear) Patlak model (i.e., the SD of the estimator meets the CRB tightly). The average SDs for the tumor *v*_p_ are approximately on the same order of magnitude irrespective of whether the Patlak or extended Tofts model is used. Average SDs for *K* ^trans^, however, are about one order of magnitude lower for the Patlak model compared to the extended Tofts estimates across all sampling patterns. Although the exact values for SDs of TK parameters estimated according to the Patlak and extended Tofts model differ, the trends and relative rankings are identical for higher undersampling rates (i.e., R ≥ 9). For the Patlak model, the relative rankings of sampling patterns are identical irrespective of the TK parameter and whether low SD or maximum coefficient is of concern. Lattice sampling with uniform density outperformed all other sampling patterns in this comparison at R ≥ 9. This is in contrast to estimation of *K* ^trans^ of the extended Tofts model where randomized Golden Angle pseudo‐radial sampling achieves lowest maximum coefficient of variation.

## 4 DISCUSSION

This study compared eight different Cartesian sampling strategies for high‐resolution whole brain DCE‐MRI with tracer‐kinetic model constraints. Uniform density, lattice‐based sampling provided the best performance overall. Lattice‐based sampling with higher density at the k‐space center led to a loss in precision of up to 5%. Differences in averaged SD to random sampling with uniform and variable density were up to 19% for *v*_p_ and 10% for *K* ^trans^ at undersampling factors of up to R = 25. TRICKS as well as pseudo radial sampling with Golden Angle and its randomized derivative exhibited substantially higher SDs at R > 5 that incurred a loss relative to uniform lattice sampling of no more than 10% in maximum coefficient of variation for *K* ^trans^ up to R = 25. At R = 4, randomized Golden Angle radial sampling achieved the lowest average SDs and maximum coefficient of variation for extended Tofts *K* ^trans^ in the tumor ROI.

We observed several effects for the different sampling patterns in the spatial maps of CRB‐predicted SDs: For the simulated 8‐channel head array all spatial maps showed central enhancement. As a consequence, measurements of tumors located in the center of the brain (e.g., pituitary adenomas) will be associated with higher measurement uncertainty because of noise rather than tumors at the brain boundary (e.g., meningioma). TRICKS sampling displayed swirl‐like pattern in the CRB‐predicted SD maps whereas lattice‐based sampling resulted in stripes of enhanced SD. Similar to g‐factor maps, CRBs do no provide a way to directly explain the sources of noise amplification arising from correlations in (k,t) space and arbitrary undersampling.^27^ The origins of these effects and patterns remain subject to future research.

We found the SDs for *K* ^trans^ obtained with the Patlak model to be about one order of magnitude lower compared to those of the extended Tofts model. This suggests a substantial performance boost if the simpler Patlak model is used when appropriate. We anticipate that this could be taken advantage of by incorporating model selection criteria, such as the Akaike Information Criterion, the Bayesian Information Criterion, or F‐tests,^53^ into a recently published framework by Guo et al^54^ This framework allows fitting of TK parameters to raw k‐space data, while providing the necessary flexibility in the TK modelling to incorporate such model selection.

Other prominent sources of error in DCE‐MRI are estimation errors in the auxiliary input parameters such as pre‐contrast T_1_ and the arterial or vascular input functions that propagate into the TK parameter estimates. Bane et al^55^ found average precision error measured as CV in native T_1_ maps of about 20% on 3T scanners using the commonly deployed Variable Flip‐Angle (VFA) Method. Applying first order error propagation analysis, such error would propagate (super‐) proportionally into the CV of TK parameters.^8^, ^56^, ^57^ Huang et al^5^ found a maximum CV of 70% in *K* ^trans^ in prostate DCE‐MRI because of AIF detection, whereas Klawer et al^58^ showed that the average CV of *K* ^trans^ because of inaccuracy in AIF estimation can be reduced to about 12% with the proposed AIF estimation in the complex plane. Accurate and precise estimation of the peak contrast during the first pass of the arterial input function remains an open and challenging problem^59^ that leads to (super‐) proportional errors in estimated TK parameters.^8^ Regarding these additional error sources, a choice of a sampling pattern based on the presented noise statistics during the dynamic part of a brain tumor DCE‐MRI exam could likely be outweighed by other sources of error.

Although the main focus of this study is DCE‐MRI for brain tumors, this study could potentially inform DCE‐MRI protocols for age‐related neurological diseases such as Alzheimer's disease. These DCE‐MRI exams typically observe very slow leakage with *K* ^trans^ on the order of approximately 10^−4^ to 10^−3^ min^−1^.^9^, ^60^ This low leakage regime requires high precision protocols to compare subject versus control cohorts.^60^ Previous work demonstrated that such specialized protocols can be obtained by long baseline acquisitions in conjunction with measures to achieve many high‐quality samples of the CTCs (i.e., long acquisition times), high temporal sampling rates, or by trading low temporal resolution (~60 s) for noise‐reduced, averaged samples.^9^, ^60^ Although the problem of patient‐specific AIF estimation remains, it may especially be advantageous to opt for the noise‐optimal (k,t) sampling strategy in this low leakage regime.

This work assumed a known AIF, but as mentioned previously, an evaluation of different sampling strategies depends critically on the imaging assumptions. In practical settings, the AIF is not always considered to be known. Although population‐based AIFs can be used to reduce measurement uncertainty,^42^, ^61^ benefits have been found in measuring patient‐specific AIFs because of large variability of AIFs across patients.^62^ In this case, the sampling pattern has to accommodate the needs to estimate the bolus arrival time and patient‐specific AIF on top of TK parameter estimation.^10^ The present results indicate design freedom in the choice of sampling pattern when TK parameter estimation is concerned, which can be used to tailor sampling patterns toward their ability to detect bolus arrival and arterial input functions.^63^ Fortunately, the same analysis (Monte‐Carlo analysis, CRB analysis) is also possible if the AIF is assumed to be unknown. This promises to be an interesting extension for future work.

In a similar vein, DCE MRI may suffer from motion corruption (e.g., in the case of liver or breast imaging). Previous work in dynamic MRI has addressed this issue by design of sampling patterns that enable motion to be estimated from the acquired data in (k,t) space for subsequent correction.^64^ In case of expected dominant motion corruption, one could opt for a sampling scheme that lends itself well to motion correction without paying too high a noise penalty.

This work focused on Cartesian sampling patterns only. These types of sampling pattern do not require any form of gridding which is common in radial, spiral, kooshball, or cone sampling for example. Gridding introduces noise correlations between different k‐space locations. Because such noise correlations were not part of the current study, these non‐Cartesian sampling strategies require further investigation to assess their performance.

The framework in this study consists of a digital reference object and Cramér‐Rao analysis to compare various (k,t) sampling strategies. We further use Monte Carlo simulations to demonstrate that direct fitting of TK parameters to k‐space data remain within 2.5 times the CRB predicted SDs for *v*_p_ and *K* ^trans^ under no assumptions beyond knowledge of the AIF and the nonlinear 3‐parameter extended Tofts tracer kinetic model. Implementation and testing of all varieties of compressed sensing and constrained reconstruction approaches for parameter estimation in DCE MRI is beyond the scope of this study. This present methodology could, however, be used to test the statistical variance of additional acquisition and reconstruction combinations, potentially including constrained reconstruction algorithms not included in this work.^65^, ^66^, ^67^ This framework can readily be translated to DCE exams for other body parts such as breast, prostate, or liver by creating the respective anatomically realistic reference objects and measuring coil sensitivity maps. This could help to elicit MRI exams that benefit from a specific choice of sampling pattern. This work follows a current trend in quantitative MRI with the goal of estimating parameters of known physiological models with low variance. Other examples include blood volume or flow and transit times in dynamic susceptibility contrast MRI or arterial spin labeling.

## 5 CONCLUSIONS

We have carried out an evaluation of the impact of (k,t) sampling scheme on the SDs of TK parameters in sparse brain tumor DCE‐MRI using digital reference objects. This approach is applicable when a realistic DRO is available, when an appropriate kinetic model is known, and when the AIF, coil sensitivities, and noise properties are known. We found that lattice sampling produced the lowest SDs overall for the estimation of TK parameters with the Patlak and extended Tofts model at undersampling rates above 9, and zone‐based sampling produced the highest SDs overall. This trend was consistent for the maximum coefficient of variation in the estimation of *v*_p_ of the Patlak and extended Tofts model and *K* ^trans^ of the Patlak model. For *K* ^trans^ of the extended Tofts model, randomized golden angle pseudo‐radial sampling achieved lowest maximum coefficient of variation. Interestingly, we found no substantial benefit of variable over uniform density for both lattice and pseudo‐random sampling. Furthermore, lattice and random sampling were nearly equivalent; with random sampling leading to at most 19% higher TK parameter SDs, which is smaller than many other known sources of variation in vivo.^5^, ^8^, ^55^, ^59^ This suggests that among the lattice and random sampling variants, selection of 1 specific sampling scheme should be guided by indirect benefits such as estimation of the patient‐specific AIF, estimation of coil sensitivity maps, and/or detection and compensation of patient motion.

## Supporting information


**FIGURE S1** Estimator performance as a function of SNR for both *v*_p_ (left column) and *K* ^trans^ (right column) of the Patlak model: comparison of SDs predicted by CRB with SDs computed by Monte‐Carlo simulation with conventional (conv) and direct reconstruction with (WLS) and without (OLS) accounting for correlated, non‐isotropic noise for uniform random sampling with R = 4 and uniform density undersampling
**FIGURE S2** Comparison of sampling patterns as a function of undersampling factor for the estimation of *v*_p_ (left column) and *K* ^trans^ (right column) with the Patlak model. CRB‐predicted SDs averaged over brain tumor regions are shown in the top row and maximum coefficient of variation, i.e., SD divided by true parameter, in the bottom row. For all sampling patterns except Keyhole sampling, the averaged SDs for both TK parameters scale approximately with the square root of the undersampling factor at low undersampling factors. As the undersampling factor is increased, the scaling behavior deviates from this rule indicating an increased interaction between the undersampling and the coil geometry
**FIGURE S3** Relative comparison of sampling patterns as a function of undersampling factor for the estimation of *v*_p_ (left column) and *K* ^trans^ (right column) with the Patlak model. Relative increase in SD bounds averaged across the tumor ROI (top row) and maximum coefficients of variation across the tumor ROI (bottom row) are shown with respect to figures of lattice with UD. Although random sampling with uniform and variable density perform best at R = 4, lattice sampling leads to lowest average SD for both *v*_p_ and *K* ^trans^ compared to uniform sampling at higher undersampling factors. For both lattice and random sampling, the uniform density variants perform comparable to their variable density counterparts.
**FIGURE S4** Patlak *v*_p_ SD bounds within brain tumor regions of interest, as a function of sampling strategy and undersampling factor. Fully sampled data is chosen as reference. Top row left to right: Keyhole sampling, TRICKS, Golden Angle radial and randomized Golden Angle radial. Bottom row left to right: lattice with uniform (UD) and variable density (VD), random sampling with uniform (UD) and variable density (VD). Different undersampling factors are indicated by color. Bounds are shown for SNR = 30
**FIGURE S5** Patlak *K* ^trans^ SD bounds within brain tumor regions of interest, as a function of sampling strategy and undersampling factor. Fully sampled data is chosen as reference. Top row left to right: Keyhole sampling, TRICKS, Golden Angle radial and randomized Golden Angle radial. Bottom row left to right: lattice with uniform (UD) and variable density (VD), random sampling with uniform (UD) and variable density (VD). Different undersampling factors are indicated by color. Bounds are shown for SNR = 30
**FIGURE S6** Visualization of Patlak *v*_p_ parameter SDs predicted by CRB for different sampling patterns at undersampling factor 16 for the first DRO in Figure 1. Top row left to right: Keyhole sampling, TRICKS, Golden Angle radial and randomized Golden Angle radial. Bottom row left to right: lattice with uniform (UD) and variable density (VD), random sampling with uniform (UD) and variable density (VD). All sampling patterns show central enhancement of SD bounds for both TK parameters
**FIGURE S7** Visualization of Patlak *K* ^trans^ parameter SDs predicted by CRB for different sampling patterns at undersampling factor 16 for the first DRO in Figure 1. Top row left to right: Keyhole sampling, TRICKS, Golden Angle radial and randomized Golden Angle radial. Bottom row left to right: lattice with uniform (UD) and variable density (VD), random sampling with uniform (UD) and variable density (VD). All sampling patterns show central enhancement of SD bounds for both TK parameters
**FIGURE S8** Visualization of Patlak *K* ^trans^ SDs predicted by CRB for different sampling patterns at undersampling factor 16 for the tumor region of the first DRO in Figure 1. CRB accounts for measured coil sensitivities from an 8‐channel head array
**FIGURE S9** The SNR map for the simulated 8‐channel head array with measured coil sensitivities and noise covariance matrix. The SNR map is computed for a hypothetical object with uniform signal across the whole field‐of‐view.^74^, ^75^ The SNR map shows a decrease of SNR in the center of the field‐of‐view characteristic for head array coils.^75^ The SNR map is mildly asymmetric with respect to the center of the FOV
**TABLE S1** Simulation parameters for each tissue type in the Digital Reference Objects. Parameters for tissue types are taken from literature values^1^, ^38^, ^42^, ^60^, ^69^, ^70^, ^71^, ^72^, ^73^ and chosen to visually match magnitude images in clinical brain tumor DCE exams at our institution (3T, HD23, GE Healthcare, Waukesha, WI)

**VIDEO S1** Illustration of data sampling strategies for 9‐fold undersampling. Shown are the *k_y_*, *k_z_* phase encoding planes for different time frames. White dots indicate phase encodes that are acquired. Black indicate phase encodes that are not acquired. Left‐to‐right: Keyhole, TRICKS, lattice with uniform density, lattice with variable density, random with uniform density, and random with variable density, pseudo‐radial with Golden Angle increments (GAR) and randomized pseudo‐radial with Golden Angle increments (RGAR). Each sampling pattern had a fully sampled first time frame, and varied for all subsequent time frames
