# Supplementary material for: Impact of (k,t) sampling on DCE MRI tracer kinetic parameter estimation in digital reference objects
Source: Magn Reson Med. 2019 Oct 12;83(5):1625–39. doi: 10.1002/mrm.28024 (PMC6982604; doi:10.1002/mrm.28024)
Supplement: Supplementary file 1 — FIGURE S1 Estimator performance as a function of SNR for both v p (left column) and K trans (right column) of the Patlak model: comparison of SDs predicted by CRB with SDs computed by Monte‐Carlo simulation with conventional (conv) and direct reconstruction with (WLS) and without (OLS) accounting for correlated, non‐isotropic noise for uniform random sampling with R = 4 and uniform density undersampling FIGURE S2 Comparison of sampling patterns as a function of undersampling factor for the estimation of v p (left column) and K trans (right column) with the Patlak model. CRB‐predicted SDs averaged over brain tumor regions are shown in the top row and maximum coefficient of variation, i.e., SD divided by true parameter, in the bottom row. For all sampling patterns except Keyhole sampling, the averaged SDs for both TK parameters scale approximately with the square root of the undersampling factor at low undersampling factors. As the undersampling factor is increased, the scaling behavior deviates from this rule indicating an increased interaction between the undersampling and the coil geometry FIGURE S3 Relative comparison of sampling patterns as a function of undersampling factor for the estimation of v p (left column) and K trans (right column) with the Patlak model. Relative increase in SD bounds averaged across the tumor ROI (top row) and maximum coefficients of variation across the tumor ROI (bottom row) are shown with respect to figures of lattice with UD. Although random sampling with uniform and variable density perform best at R = 4, lattice sampling leads to lowest average SD for both v p and K trans compared to uniform sampling at higher undersampling factors. For both lattice and random sampling, the uniform density variants perform comparable to their variable density counterparts. FIGURE S4 Patlak v p SD bounds within brain tumor regions of interest, as a function of sampling strategy and undersampling factor. Fully sampled data is chosen as reference. To [file MRM-83-1625-s001.docx]

Supplemental Material to

Impact of (k,t) sampling on DCE MRI

tracer kinetic parameter estimation in digital reference objects

Yannick Bliesener^1*^, Sajan G. Lingala^1^, Justin P. Haldar^1^, and Krishna S. Nayak^1^

*^1^Ming Hsieh Department of Electrical and Computer Engineering, University of Southern California, Los Angeles, California, USA*

**Supplemental Material**

|  | **Tissue** | $\boldsymbol{\rho}$ | **T_10_ [s]** | **v_p_ [%]** | **K^trans^ [min^-1^]** | **v_e_ [%]** |
| --- | --- | --- | --- | --- | --- | --- |
| **0** | Background | 0 |  |  |  |  |
| **1** | CSF | 1 | 2.750 | 0 | 0 |  |
| **2** | Gray Matter | 1 | 1.820 | 5 | 0.0008 | 20 |
| **3** | White Matter | 1 | 1.084 | 2 | 0.002 | 20 |
| **4** | Fat | 1 | 0.471 | 0 | 0 |  |
| **5** | Muscle | 1 | 1.400 | 0 | 0 |  |
| **6** | Skull | 1 | - | 0 | 0 |  |
| **7** | Vessel | 1 | 1.932 | 60 | 0 |  |
| **8** | Bone Marrow | 0.5 | 0.471 | 0 | 0 |  |
| **9** | Tumor | 1 | 1.000 | 0.16-12.25 | 0.03-0.42 |  |

**Supporting Information Table S1:** Simulation parameters for each tissue type in the Digital Reference Objects. Parameters for tissue types are taken from literature values (1,38,42,58,67–71) and chosen to visually match magnitude images in clinical brain tumor DCE exams at our institution (3 Tesla, HD23, GE Healthcare, Waukesha, WI).


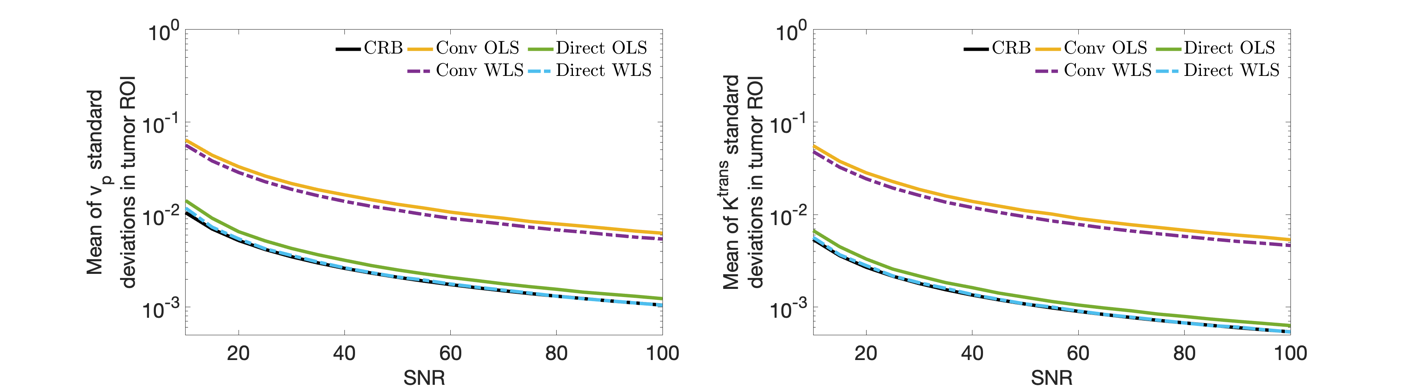


**Supporting Information Figure S1:** Estimator performance as a function of SNR for both *v*_p_ (left column) and *K*^trans^ (right column) of the Patlak model: Comparison of standard deviations predicted by CRB with standard deviations computed by Monte-Carlo simulation with conventional (conv) and direct reconstruction with (WLS) and without (OLS) accounting for correlated, non-isotropic noise for uniform random sampling with R = 4 and uniform density undersampling.


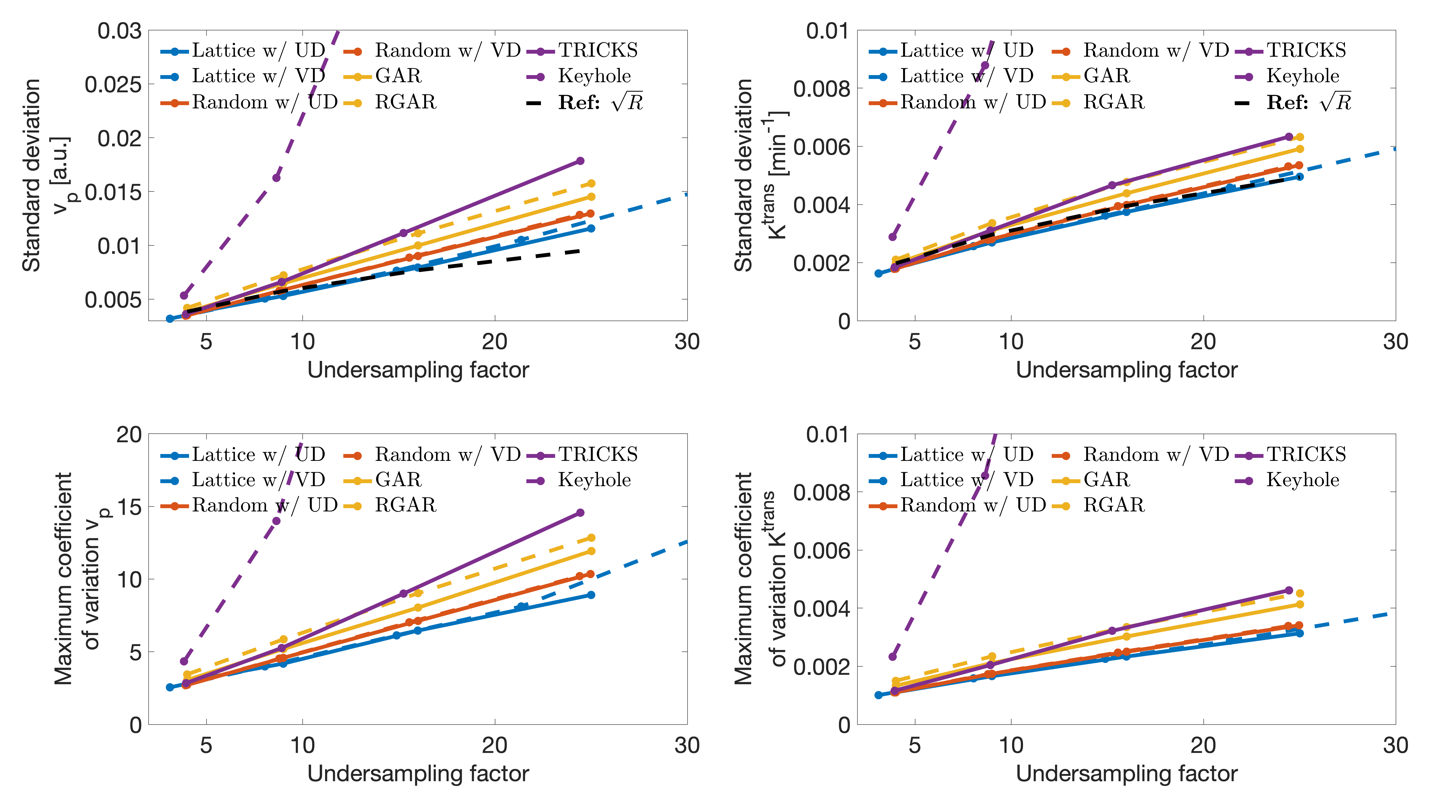


**Supporting Information Figure S2:** Comparison of sampling patterns as a function of undersampling factor for the estimation of *v*_p_ (left column) and *K*^trans^ (right column) with the Patlak model. CRB-predicted standard deviations averaged over brain tumor regions are shown in the top row and maximum coefficient of variation, i.e., standard deviation divided by true parameter, in the bottom row. For all sampling patterns except Keyhole sampling, the averaged standard deviations for both TK parameters scale approximately with the square root of the undersampling factor at low undersampling factors. As the undersampling factor is increased, the scaling behavior deviates from this rule indicating an increased interaction between the undersampling and the coil geometry.


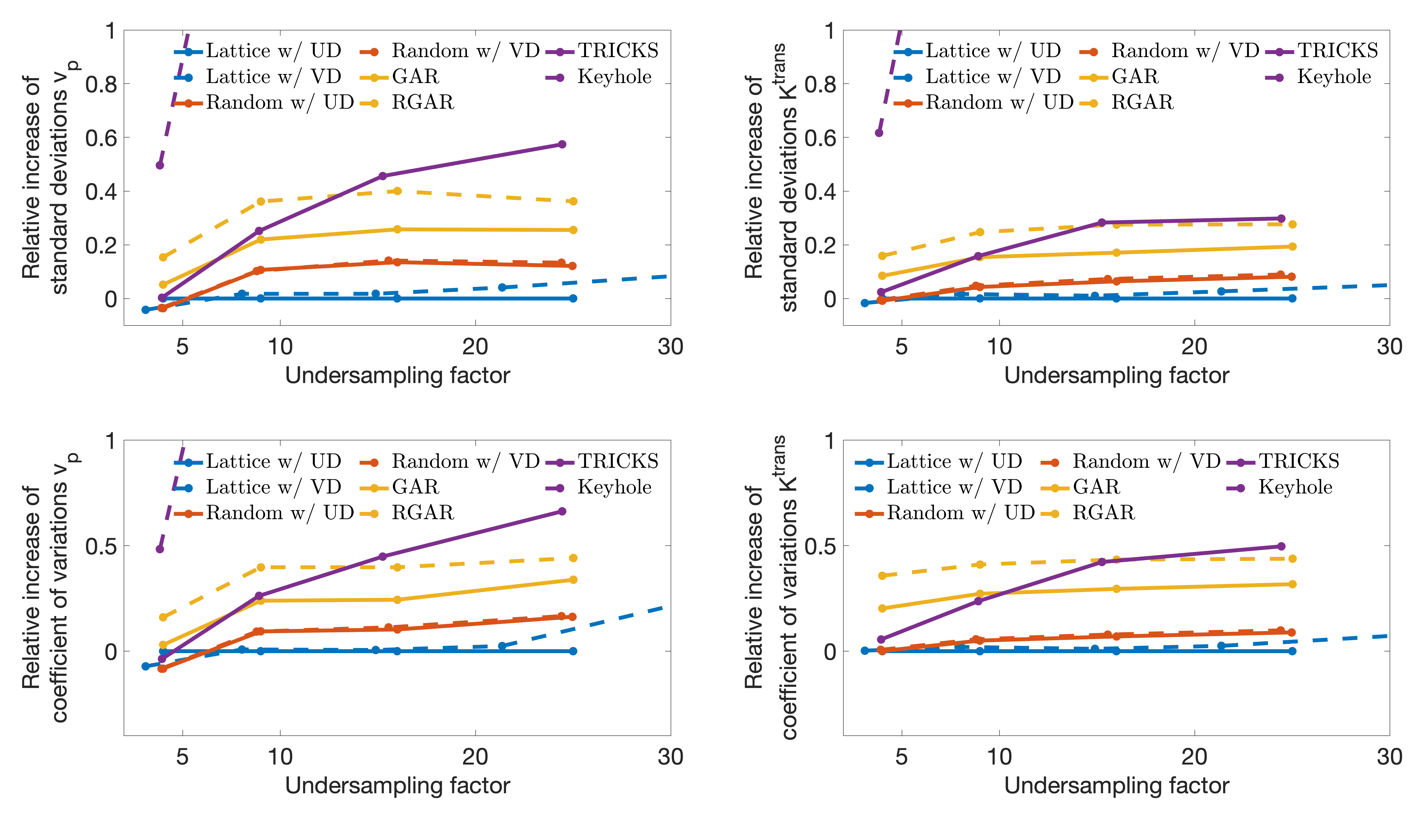


**Supporting Information Figure S3:** Relative comparison of sampling patterns as a function of undersampling factor for the estimation of *v*_p_ (left column) and *K*^trans^ (right column) with the Patlak model. Relative increase in standard deviation bounds averaged across the tumor ROI (top row) and maximum coefficients of variation across the tumor ROI (bottom row) are shown w.r.t. figures of lattice with UD. While random sampling with uniform and variable density perform best at R = 4, lattice sampling leads to lowest average standard deviation for both *v*_p_ and *K*^trans^ compared to uniform sampling at higher undersampling factors. For both lattice and random sampling, the uniform density variants perform comparable to their variable density counterparts.


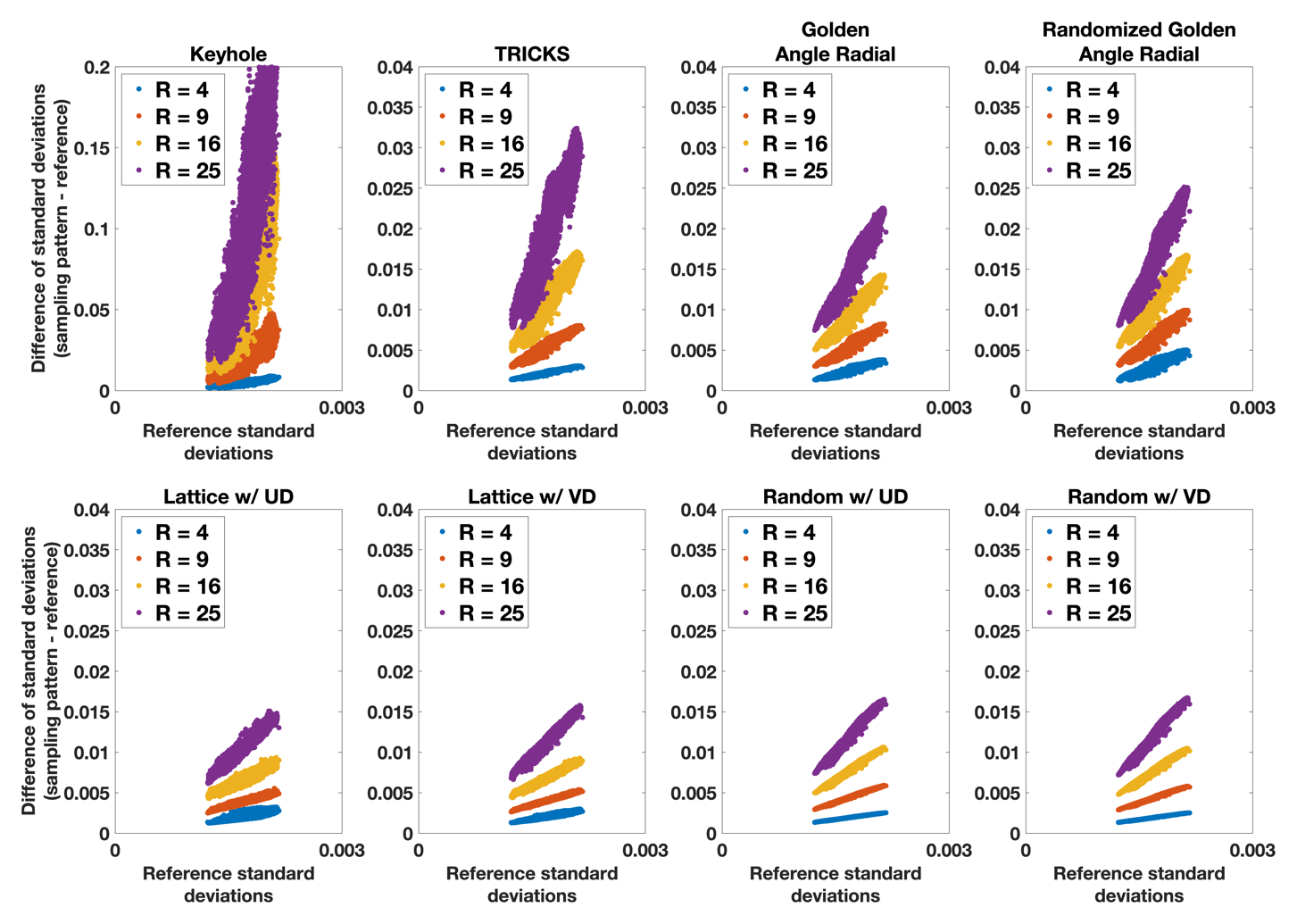


**Supporting Information Figure S4:** Patlak *v*_p_ standard deviation bounds within brain tumor regions of interest, as a function of sampling strategy and undersampling factor. Fully sampled data is chosen as reference. Top row left to right: Keyhole sampling, TRICKS, golden angle radial and randomized golden angle radial. Bottom row left to right: Lattice with uniform (UD) and variable density (VD), random sampling with uniform (UD) and variable density (VD). Different undersampling factors are indicated by color. Bounds are shown for SNR = 30.


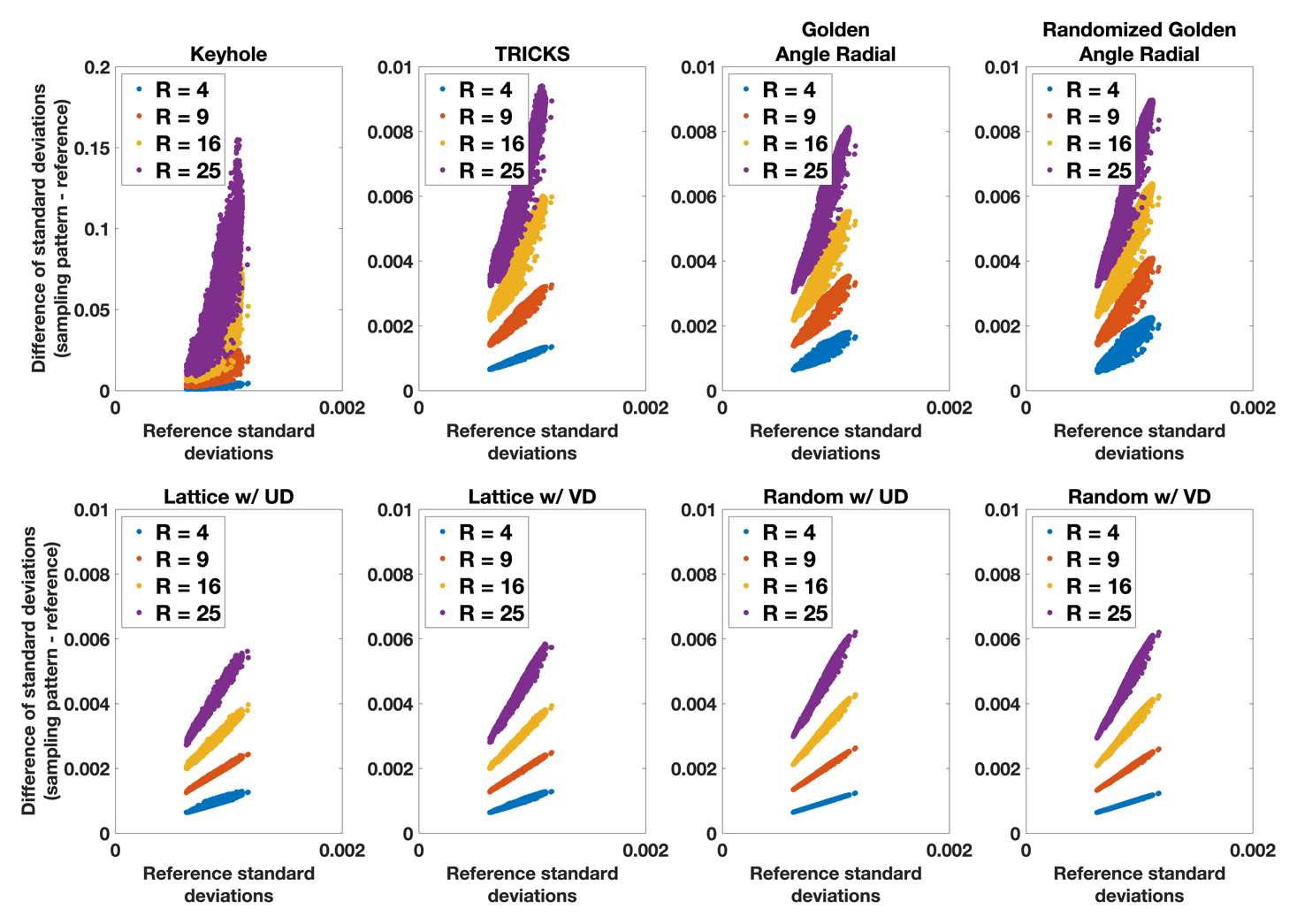


**Supporting Information Figure S5:** Patlak *K*^trans^ standard deviation bounds within brain tumor regions of interest, as a function of sampling strategy and undersampling factor. Fully sampled data is chosen as reference. Top row left to right: Keyhole sampling, TRICKS, golden angle radial and randomized golden angle radial. Bottom row left to right: Lattice with uniform (UD) and variable density (VD), random sampling with uniform (UD) and variable density (VD). Different undersampling factors are indicated by color. Bounds are shown for SNR = 30.


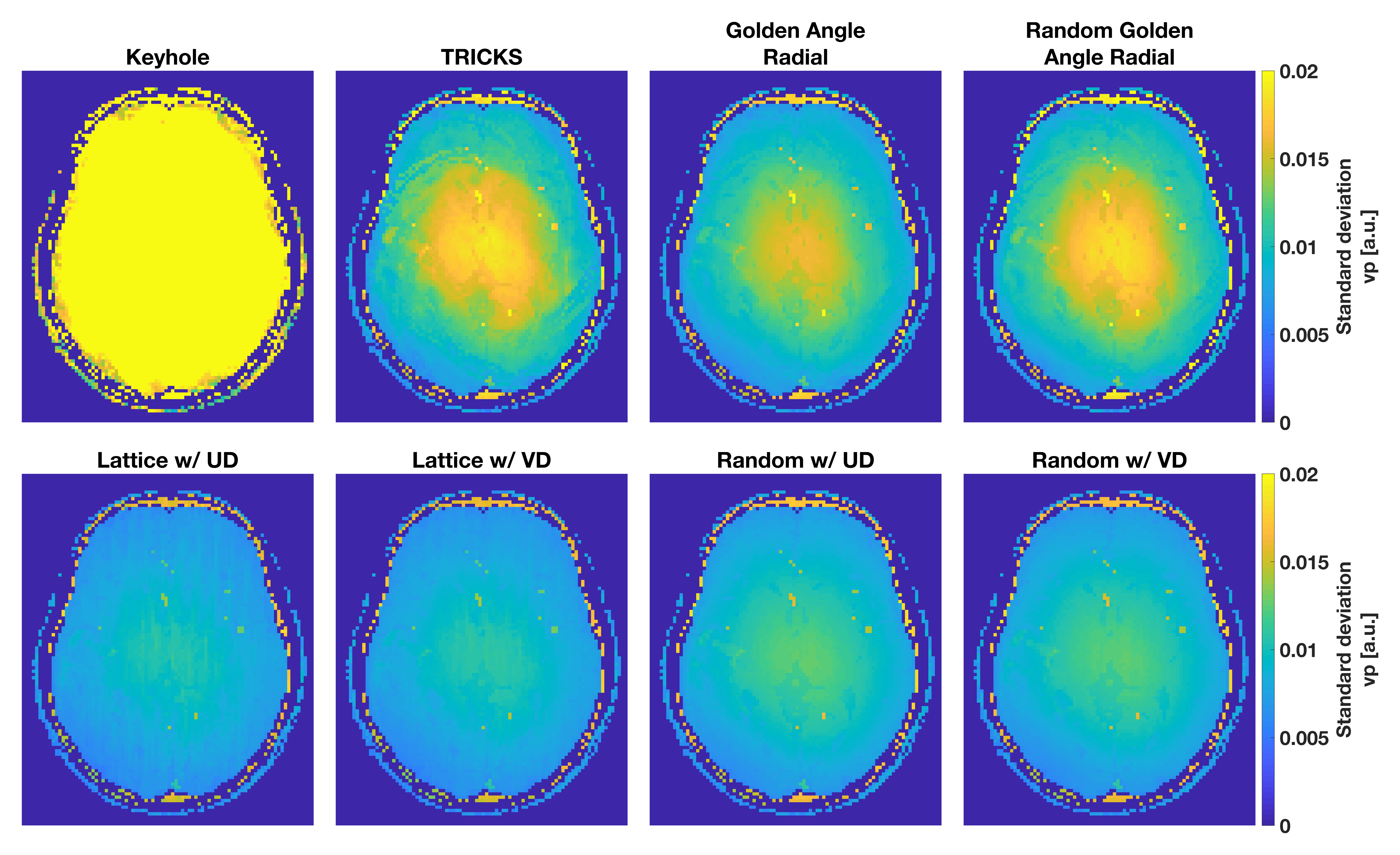


**Supporting Information Figure S6:** Visualization of Patlak *v*_p_ parameter standard deviations predicted by CRB for different sampling patterns at undersampling factor 16 for the first DRO in Figure 1. Top row left to right: Keyhole sampling, TRICKS, golden angle radial and randomized golden angle radial. Bottom row left to right: Lattice with uniform (UD) and variable density (VD), random sampling with uniform (UD) and variable density (VD). All sampling patterns show central enhancement of standard deviation bounds for both TK parameters.


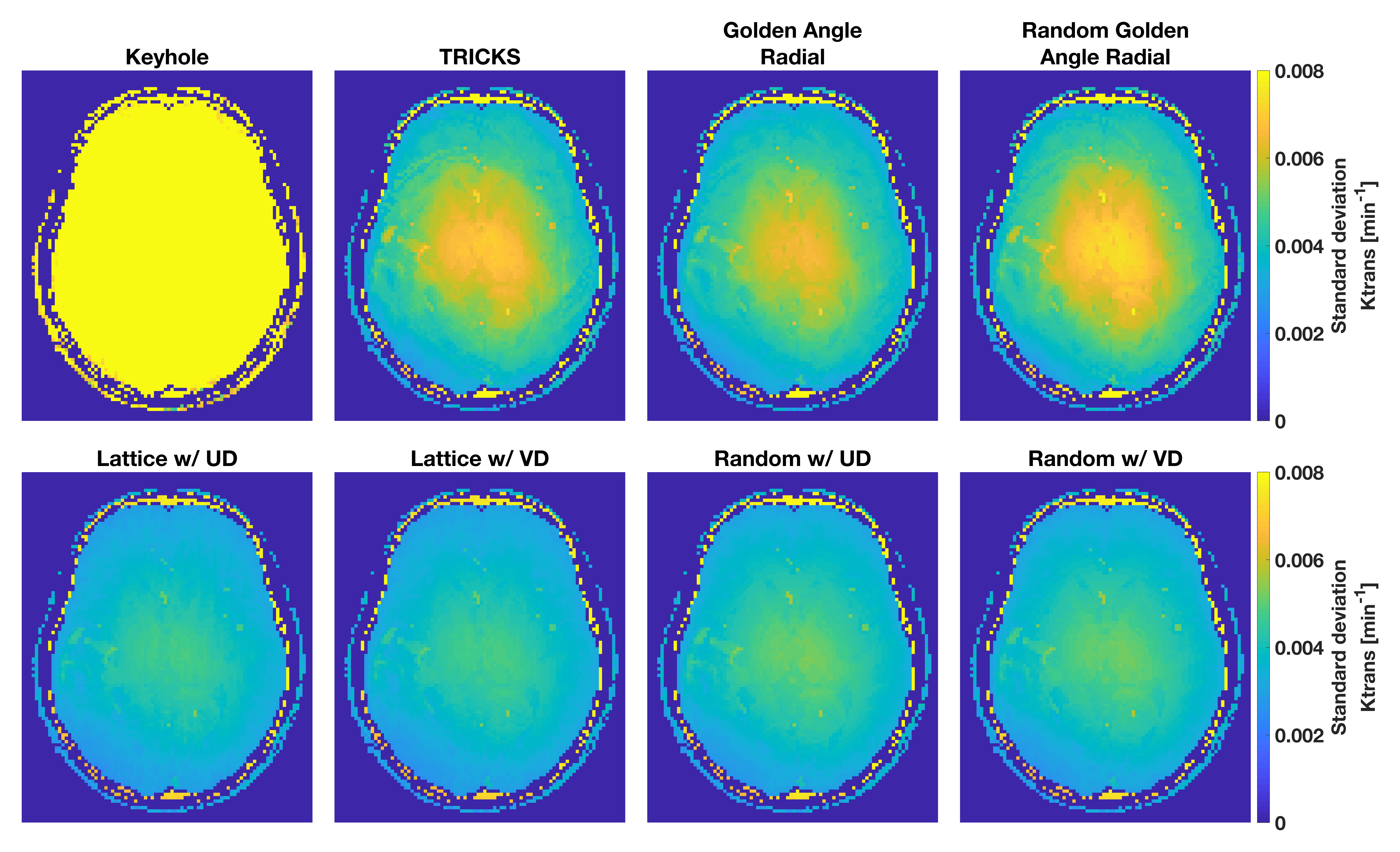


**Supporting Information Figure S7:** Visualization of Patlak *K*^trans^ parameter standard deviations predicted by CRB for different sampling patterns at undersampling factor 16 for the first DRO in Figure 1. Top row left to right: Keyhole sampling, TRICKS, golden angle radial and randomized golden angle radial. Bottom row left to right: Lattice with uniform (UD) and variable density (VD), random sampling with uniform (UD) and variable density (VD). All sampling patterns show central enhancement of standard deviation bounds for both TK parameters.


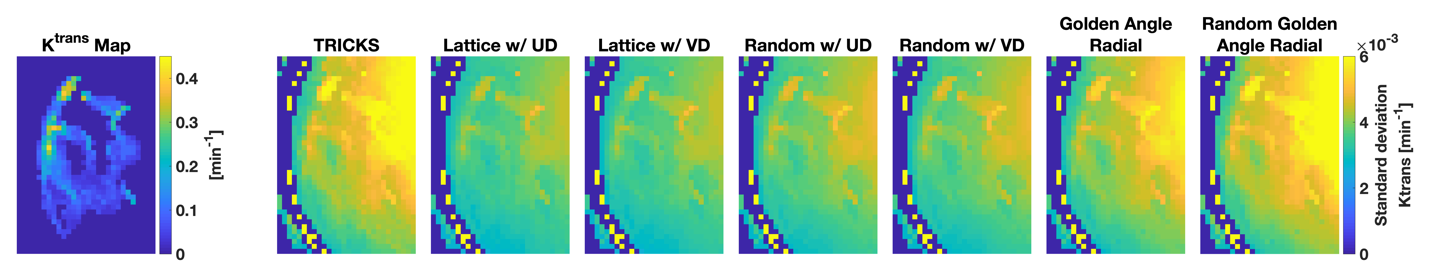


**Supporting Information Figure S8:** Visualization of Patlak *K*^trans^ standard deviations predicted by CRB for different sampling patterns at undersampling factor 16 for the tumor region of the first DRO in Figure 1. CRB accounts for measured coil sensitivities from an 8-channel head array.


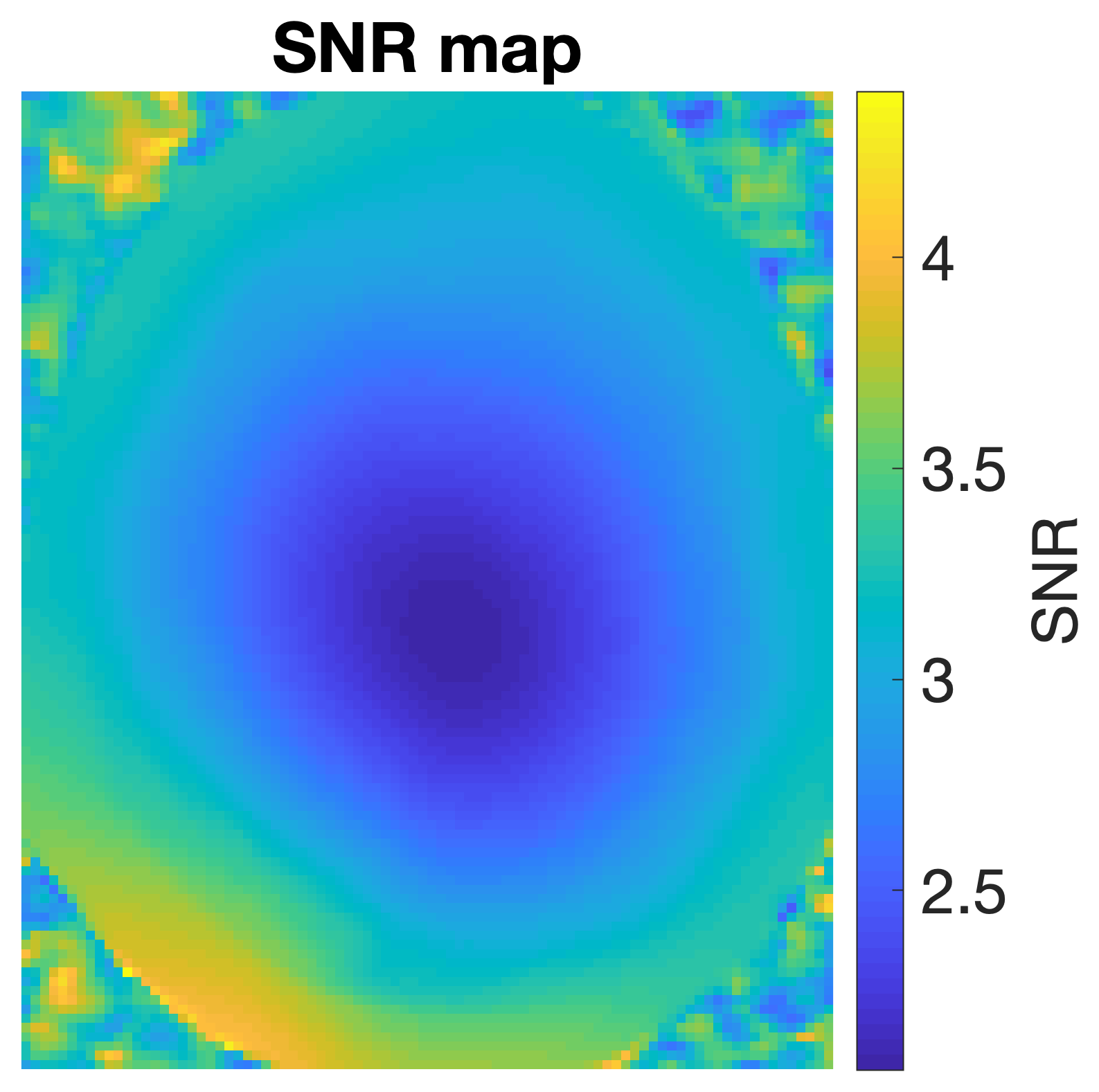


**Supporting Information Figure S9:** The SNR map for the simulated 8-channel head array with measured coil sensitivities and noise covariance matrix. The SNR map is computed for a hypothetical object with uniform signal across the whole field-of-view (72,73). The SNR map shows a decrease of SNR in the center of the field-of-view characteristic for head array coils (73). The SNR map is mildly asymmetric with respect to the center of the FOV.

**Supporting Information Video S1:** Illustration of data sampling strategies for nine-fold undersampling. Shown are the k_y_,k_z_ phase encoding planes for different time frames. White dots indicate phase encodes that are acquired. Black indicate phase encodes that are not acquired. Left-to-right: Keyhole, TRICKS, Lattice with uniform density, Lattice with variable density, Random with uniform density, and Random with variable density, pseudo-radial with Golden Angle increments (GAR) and randomized pseudo-radial with Golden Angle increments (RGAR). Each sampling pattern had a fully-sampled first time frame, and varied for all subsequent time frames.
